# *Lactobacillus plantarum* TWK10 Attenuates Aging-Associated Muscle Weakness, Bone Loss, and Cognitive Impairment by Modulating the Gut Microbiome in Mice

**DOI:** 10.3389/fnut.2021.708096

**Published:** 2021-10-13

**Authors:** Chia-Chia Lee, Yi-Chu Liao, Mon-Chien Lee, Kun-Ju Lin, Han-Yin Hsu, Shiou-Yun Chiou, San-Land Young, Jin-Seng Lin, Chi-Chang Huang, Koichi Watanabe

**Affiliations:** ^1^Culture Collection & Research Institute, SYNBIO TECH INC., Kaohsiung, Taiwan; ^2^Institute of Population Health Sciences, National Health Research Institutes, Miaoli, Taiwan; ^3^Graduate Institute of Sports Science, National Taiwan Sport University, Taoyuan, Taiwan; ^4^Department of Nuclear Medicine, Linkou Chang Gung Memorial Hospital, Taoyuan, Taiwan; ^5^Department of Medical Imaging and Radiological Sciences, Chang Gung University, Taoyuan, Taiwan; ^6^Bioresource Collection and Research Center, Food Industry Research and Development Institute, Hsinchu, Taiwan; ^7^Department of Animal Science and Technology, National Taiwan University, Taipei, Taiwan

**Keywords:** *Lactobacillus plantarum* TWK10, aging, sarcopenia, gut microbiota, muscle, memory, osteoporosis

## Abstract

In humans, aging is characterized by the progressive decline in biological, physiological, and psychological functions, and is a major risk factor in the development of chronic diseases. Therefore, the development of strategies aimed at attenuating aging-related disorders and promoting healthy aging is critical. In a previous study, we have demonstrated that *Lactobacillus plantarum* TWK10 (TWK10), a probiotic strain isolated from Taiwanese pickled cabbage, improved muscle strength, exercise endurance, and overall body composition in healthy humans. In this study, the effect of TWK10 on the progression of age-related impairments was investigated in mice. We found that TWK10 not only enhanced muscle strength in young mice, but also prevented the aging-related loss of muscle strength in aged mice, which was accompanied by elevated muscle glycogen levels. Furthermore, TWK10 attenuated the aging-associated decline in learning and memory abilities, as well as bone mass. Further analyses of gut microbiota using next-generation sequencing (NGS) of the 16S rRNA gene showed that the pattern of gut microbial composition was clearly altered following 8 weeks of TWK10 administration. TWK10-treated mice also experienced an increase in short-chain fatty acid (SCFA)-producing bacteria and higher overall levels of gut SCFA. Furthermore, TWK10 administration to some extent reversed the aging-associated accumulation of pathogenic bacterial taxa. In conclusion, TWK10 could be viewed as a potential therapeutic agent that attenuates aging-related disorders and provides health benefits by modulating the imbalance of gut microbiota.

## Introduction

Aging is a progressive process associated with negative changes in the physical performance, body composition, learning and memory, social and psychological responses, joints, and metabolic regulation. Aging-associated decline in the functions of tissues and organs represents a major risk factor in the development of chronic disease (1). Aging is accompanied by a reduction in body lean mass and bone mineral density, and an increase in fat mass. Sarcopenia is generally defined as the progressive loss of skeletal muscle mass and strength that occurs with aging (2, 3). Muscle mass drops by ~3–8% per decade after age 30 and its declining rate is accelerated after age 60 (4). Muscle loss is closely correlated with an increased risk of falls and fractures, physical disability, poor quality of life, and death. Furthermore, fat mass usually increases progressively with age, and is particularly localized to the abdominal region. Accumulation of abdominal fat mass is closely related to the onset of metabolic disorders, including cardiovascular disease and diabetes (5).

Low bone mass, a condition known as osteoporosis, usually happens as a result of aging. Osteoporosis is associated with an increased risk of bone fracture and fracture-associated mortality. In humans, bone mass gradually increases and peaks in the 30s and starts to decline again in the 40s (6). Meanwhile, learning and memory start to gradually decline as early as in the 20s and 30s, with the decline becoming more prominent after reaching 60 years of age. Learning and memory impairment interferes with the daily lives of elderly individuals. Approximately 40% of people aged 60 years or older have memory impairments, and each year ~1% of them will go on to develop dementia (7, 8).

The gut microbiome plays an important role in health maintenance, and the imbalances of gut microbiota are closely related to aging (9, 10). It has been shown that elderly people have a different gut microbiome compared to younger adults. With aging, the diversity of gut microbiota appears to decline while levels of opportunistic microbes, such as members of enterobacteria and *Clostridium* spp. become more abundant (10, 11). Short-chain fatty acids (SCFAs) are produced by anaerobic intestinal bacteria as end products of dietary fiber fermentation. Butyrate, propionate, and acetate account for 90% of total SCFAs present in the colon. These metabolites have been shown to play important roles in host physiology, by modulating metabolism, gut permeability, inflammatory responses, and immune function (12–15). SCFAs also protect the host from several diseases, including colorectal cancer, inflammatory bowel disease, and diabetes (16–18). *Bacteroidetes* and *Firmicutes* are the most abundant phyla in the human gut, accounting for over 85% of microbial composition (19). In the human gut, *Bacteroidetes* members mainly produce acetate and propionate, while *Firmicutes* populations mostly produce butyrate (20). Administration of *Lactobacillus acidophilus* DDS-1 in an aging mouse model has been shown to trigger gut microbial composition shifts, leading to an improvement in the metabolic phenotype, and enhancing the production of cecal and mucosal SCFAs (21, 22). Therefore, stimulation of SCFA production and enrichment of SCFA-producing bacteria in the gut are essential for improving overall health.

Population aging is becoming a serious problem facing all humans. By 2050, the number of people aged 65 or older is expected to reach 1.5 billion globally (World Population Aging 2019 Highlights, https://digitallibrary.un.org/record/3846855). With a fast-growing elderly population, the development of strategies for attenuating aging-related disorders and promoting healthy aging, has become a crucial issue. Recent reports have highlighted the use of probiotic supplements as potential therapeutic agents to combat aging. For example, *Lactobacillus salivarius* FDB89 has been shown to promote the growth and prolong the lifespan of *Caenorhabditis elegans* (23). Lee et al. (24) also demonstrated the skin anti-aging property of *Lactobacillus plantarum* HY7714 in humans. Supplementation of *Lactococcus lactis* subsp. *lactis* strain Plasma reduced the expression of genes associated with muscle degeneration and decelerated senescence (25). Moreover, consumption of *Lactobacillus paracasei* PS23 attenuated aging-related cognitive decline and muscle loss (26, 27). Other research groups demonstrated that supplementation of *Lactobacillus* spp. prevented bone loss in an ovariectomized (OVX) induced osteoporotic mouse model (28, 29). However, the underlying mechanisms implicated as well as the impact of these probiotics on the gut microbiome remain unclear.

In our previous studies, we have demonstrated that *Lactobacillus plantarum* TWK10 (TWK10) exerted beneficial effects on body composition, particularly on muscle mass, muscle quality, and fat mass, in mice and humans (30, 31). In this study, we aimed to (i) analyze the potential role of TWK10 in attenuating the progression of aging, namely muscle loss, reduced muscle strength, cognitive deficits, body fat accumulation, and bone density loss; and (ii) investigate the impact of TWK10 on the gut microbiome.

## Materials and Methods

### Animals, Probiotics, and Study Design

Male ICR mice were purchased from BioLASCO (Charles River Licensee Corp., Yi-Lan, Taiwan). All mice were housed under humidity-controlled (65 ± 5%), temperature-controlled (24 ± 2°C) conditions, kept on a 12:12 light-dark cycle, provided with a standard laboratory diet (No. 5001; PMI Nutrition International, Brentwood, MO, USA) and distilled water *ad libitum*. All experimental procedures were approved by the Institutional Animal Care and Use Committee (IACUC) of National Taiwan Sport University, Taoyuan City, Taiwan (IACUC-10712). TWK10, a plant-derived *L*. *plantarum* strain [recently reclassified as *Lactiplantibacillus plantarum* (32)], isolated from Taiwanese pickled cabbage (33), was obtained from the SYNBIO TECH INC. Culture Collection (Kaohsiung, Taiwan). The concentration of TWK10 was adjusted to 1 × 10^9^ CFU in 200 μl PBS for administration to mice. All mice were first grouped by age into the young (age 4 months; *n* = 17) and the aged groups (age 19–22 months; *n* = 16). Next, these two groups of mice were each randomly assigned to two experimental groups: Y-Control (young mice receiving PBS, 200 μl/mouse/day) and Y-TWK10 (young mice receiving TWK10, 1 × 10^9^ CFU/mouse/day), and A-Control (aged mice receiving PBS, 200 μl/mouse/day) and A-TWK10 (aged mice receiving TWK10, 1 × 10^9^ CFU/mouse/day). During the experimental trial, body weight, food intake, and water intake were recorded on a weekly basis. Freshly voided fecal samples were also collected at baseline and at 8 weeks after treatment initiation, for microbiome analysis. Forelimb grip strength was monitored at baseline (before TWK10 administration), 4, and 8 weeks (after TWK10 administration). After 8 weeks of TWK10 administration, all mice were assessed in terms of body composition and cognitive function. The mice were then sacrificed to obtain measurements relating to tissue weight, bone quality, and muscle glycogen, and perform histological analysis.

### Forelimb Grip Strength

A low-force testing system (Model-RX-5, Aikoh Engineering, Nagoya, Japan) was used to measure the absolute forelimb grip strength of treated mice as previously described (34). Briefly, the mouse was grasped at the base of the tail and lowered vertically toward the bar. The mouse was pulled slightly backwards by the tail while its two paws (forelimbs) grasped the bar, which triggered a “counter pull.” The maximal grip force in 10 trials was recorded as absolute forelimb grip strength. Forelimb grip strength was monitored at baseline, 4, and 8 weeks after TWK10/PBS administration.

### The Morris Water Maze

The Morris water maze (MWM) was used to assess the spatial learning and memory of mice as described previously (35). Briefly, the MWM test was performed in a round pool (100 cm in diameter and 30 cm in depth) containing water (26 ± 1°C) that is colored opaque with non-toxic tempera paint. The platform and extra-mazal cues remained in the same position throughout the learning trials (day 1 and day 2) and the mice were trained to find a platform below the water surface three times per day. The water maze has three starting positions, and the mice were made to start the exercise from a different starting position each day. Maximum swim time was set at 90 s. Escape latencies of the mice on day 3 were used to examine their spatial learning and memory.

### Body Composition

A micro-CT scan (Bioscan, Washington, DC, USA) was used for the measurement of adipose and lean tissues in living mice. A micro-CT air/water phantom scan (CT QC Phantom, Mediso, Hungary) was performed regulatory for Hounsfield unit (HU) calibration. All mice were anesthetized by 1% isoflurane inhalation in the prone position with legs extended. CT imaging was performed using 180 projections per rotation with 65 kVp, 1,000 ms, 0.123 mAs exposure, and a 1:1 binning factor. Sequential transaxial images through the lower body (between the femurs and abdominal region at L1 level) were obtained. The CT projections were reconstructed with a voxel size of 0.1475 × 0.1475 × 0.1477 mm^3^. Image analysis was performed using the PMOD (version 3.7 PMOD Technologies LTD, Zurich, Switzerland) analysis software. Images were first smoothed using a Gaussian 3D filter, then segmented according to tissue density, first for total volume and then for fat volume (36). A histogram of the abdominal volume of interest from L1 to L5 was generated and the distribution was bi-modal in nature, with one mode representing adipose tissue voxels, and the other lean tissue voxels (37). We used a fixed threshold of −300 to +3,500 HU for total volume, and −200 to −50 HU for fat volume, according to the best separation method for known fat and lean tissue regions, for all mice in this study.

### Bone Quality and Serum Vitamin D Measurements

Femur bones were harvested from mice and stored in methanol until analysis. Micro-CT images of each specimen were obtained using the SkyScan 1076 micro-CT (Bruker, Kontich, Belgium). The scanning parameters were set to 50 kV, 200 μA, a rotation step of 0.5°, exposure time 2.1 s, 0.5 mm Al filter, and 9 μm/pixel scan resolution. Bone quality parameters were calculated using CT-Analyser software (Bruker). The following three-dimensional (3-D) parameters were assessed: tissue volume (TV), bone volume (BV), bone volume fraction (BV/TV), bone surface (BS), bone surface density (BS/BV), trabecular thickness (Tb.Th), trabecular spacing (Tb.Sp), trabecular number (Tb.N), trabecular pattern factor (Tb.Pf), structure model index (SMI), and bone mineral density (BMD). Serum vitamin D (1,25-(OH)_2_ vitamin D) was measured using an automated analyzer (Hitachi 7060, Hitachi, Tokyo, Japan).

### Muscle Glycogen Levels

Gastrocnemius muscles of mice were isolated and stored at −80°C until subsequent analysis. Glycogen content was measured as described previously (38). Briefly, 100 mg of muscle tissue was homogenized in 0.5 ml cold perchloric acid. The homogenate was centrifuged at 15,000 × g for 15 min at 4°C and the supernatant was collected prior to determining glycogen concentration. Levels of glycogen (mg/g muscle) were determined by commercial assay kit (Sigma-Aldrich, St. Louis, MO, USA) according to the manufacturer's instructions.

### Histological Analysis

After 8 weeks of treatment, the epididymal fat pad (EFP), interscapular brown adipose tissue (BAT), and gastrocnemius muscle were carefully removed, fixed with 10% formalin overnight, and then embedded within paraffin. Paraffin-embedded tissues were sectioned at 4 μm and stained with hematoxylin and eosin (H&E) for histological examination of the EFP and BAT using a light microscope (BX-51; Olympus, Tokyo, Japan). The cross-sectional area (CSA, μm^2^) of an adipocyte, the mean number of BAT adipocytes observed per high-power-field (HPF), and the mean relative brown area in BAT (%) after H&E staining, were measured and analyzed using ImageJ software (NIH, MD, USA).

### DNA Extraction and 16S rRNA Gene Sequence Analysis

Freshly voided fecal samples were collected from mice after 8 weeks of treatment. For extraction of bacterial genomic DNA, fecal samples were washed three times with 1.0 ml of PBS and centrifugated at 14,000 × g for 5 min. The fecal pellets were resuspended in 180 μl TE buffer containing lysozyme (final conc 10 mg/ml), and the suspension with glass beads (300 mg, 0.1 mm in diameter; Biospec, Bartesville, OK, USA) was homogenized for 30 s using a FastPrep 24 homogenizer (MP Biomedicals, USA) to ensures complete disruption of cell walls and release of the DNA molecules into solution. Bacterial genomic DNA was then extracted using the Genomic DNA Mini Kit (Geneaid, Taipei, Taiwan) according to the manufacturer's instructions. DNA concentrations were determined by spectrophotometry using a BioDrop instrument (Biochrom, Biochrom Ltd., Cambridge, UK). DNA samples were stored at −20°C until further processing. The V3–V4 region of the 16S rRNA gene was amplified using specific primers (319F: 5′-CCTACGGGNGGCWGCAG-3′ and 806R: 5′-GACTACHVGGGTATCTAATCC-3′) (39) according to the 16S Metagenomic Sequencing Library Preparation procedure (Illumina).

The amplicon pool was sequenced on the Illumina MiSeq™ sequencing platform (Illumina, San Diego, CA, USA). Raw sequence data were demultiplexed using the q2-demux plugin and minimally quality-filtered with DADA2 (40) (via q2-dada2) using the QIIME 2 2019.7 (41), with resulting amplicon sequence variants (ASVs). Taxonomy was assigned to ASVs using the q2-feature-classifier (42), the classify-sklearn naive Bayes taxonomy classifier against the SILVA database (release 132), and representative sequences with a 99% of similarity (43). Both alpha diversity (Shannon and Richness indices) and beta diversity were estimated with QIIME2, using a rarefaction of 30,000 sequences. Beta diversity analysis was performed using a non-metric multidimensional scaling (NMDS) ordination plot based on the weighted UniFrac or unweighted UniFrac distances by metaMDS function in the “vegan” package of R software (Vegan: Community Ecology Package. R package version 2.5-6). Permutational multivariate analysis of variance (PERMANOVA)/Adonis test was applied to explore the significant differences on the basis of 999 permutations with vegan package in R software. Raw sequence files supporting the findings of this article are deposited at NCBI Sequence Read Archive (SRA) database with project accession number PRJNA726848.

### Measurement of Short-Chain Fatty Acid (SCFA) Levels

Ceca of mice were harvested, frozen immediately in liquid nitrogen, and stored at −80°C until further processing. One milligram of cecal content was mixed with 10 μl 70% ethanol solution, and then homogenized with appropriate amounts of glass beads (1.0 mm in diameter; Biospec Products) by vortexing at 3,000 rpm for 10 min. Homogenized samples were centrifuged at 14,000 × g for 10 min, and the supernatants were collected and processed for fatty acid derivatization according to the method described previously (44). The derivatized supernatants were filtered using a 0.22-μm polycarbonate syringe filter (Millipore, St. Charles, MO, USA). Analysis of SCFAs was performed using high-performance liquid chromatography (HPLC) (HITACHI, Tokyo, Japan). SCFAs were separated using a C18 HTec column (NUCLEODUR, Macherey-Nagel, Düren, Germany) with a 40°C column temperature, flow rate at 1 ml/min, and detection wavelength set to 400 nm.

### Statistical Analysis

Data are expressed as mean ± SD. Statistical analyses were performed using GraphPad Prism 7.04 (GraphPad Software, San Diego, CA). For comparisons of multiple groups, data were analyzed by two-way ANOVA with a *post-hoc* Tukey-Kramer test. The Kruskal-Wallis test with a *post-hoc* Dunn's test was used for multiple non-parametric comparisons. For comparisons of two groups, the Student unpaired *t*-test and the Mann-Whitney *U*-test were used for parametric or non-parametric data analyses, respectively. The Pearson's correlation coefficient and the Spearman's correlation coefficient were used for correlation analyses between forelimb grip strength and muscle glycogen levels, and between gut microbiota abundance and aging-related phenotypic features, respectively. Differences were considered statistically significant if *P* < 0.05.

## Results

### The Effect of TWK10 on the Aging-Related Decline in Skeletal Muscle Strength

All mice in the four experimental groups (Y-Control, Y-TWK10, A-Control, and A-TWK10) were fed for 8 weeks with identical diets, and their average body weight, food, and water intake are summarized in Table 1. No significant differences were observed in the body weight, food, or water intake among the four groups.

**Table 1:** General characteristics of the animals.

	**Group**			
	**Y-Control**	**Y-TWK10**	**A-Control**	**A-TWK10**
	*n* = 8	*n* = 9	*n* = 9	*n* = 7
Age (month)	4	4	19–22	19–22
Basal body weight (g)	41.1 ± 3.0^a^	43.4 ± 1.6^a^	44.0 ± 3.8^a^	44.2 ± 3.4^a^
Final body weight (g)	44.1 ± 3.2^a^	48.0 ± 2.4^a^	48.4 ± 4.2^a^	48.5 ± 6.0^a^
Food intake [(g/mouse)/day]	7.6 ± 0.7^a^	7.8 ± 0.7^a^	7.6 ± 1.1^a^	7.9 ± 2.2^a^
Water intake [(ml/mouse)/day]	14.3 ± 3.0^a^	14.0 ± 3.2^a^	13.4 ± 1.3^a^	13.5 ± 2.8^a^

*Data are represented as mean ± SD. Statistical differences among groups were analyzed by Tukey-Kramer test. Within a row, same superscript letter (a) indicates no significant difference, P > 0.05*.

**Figure 1:**
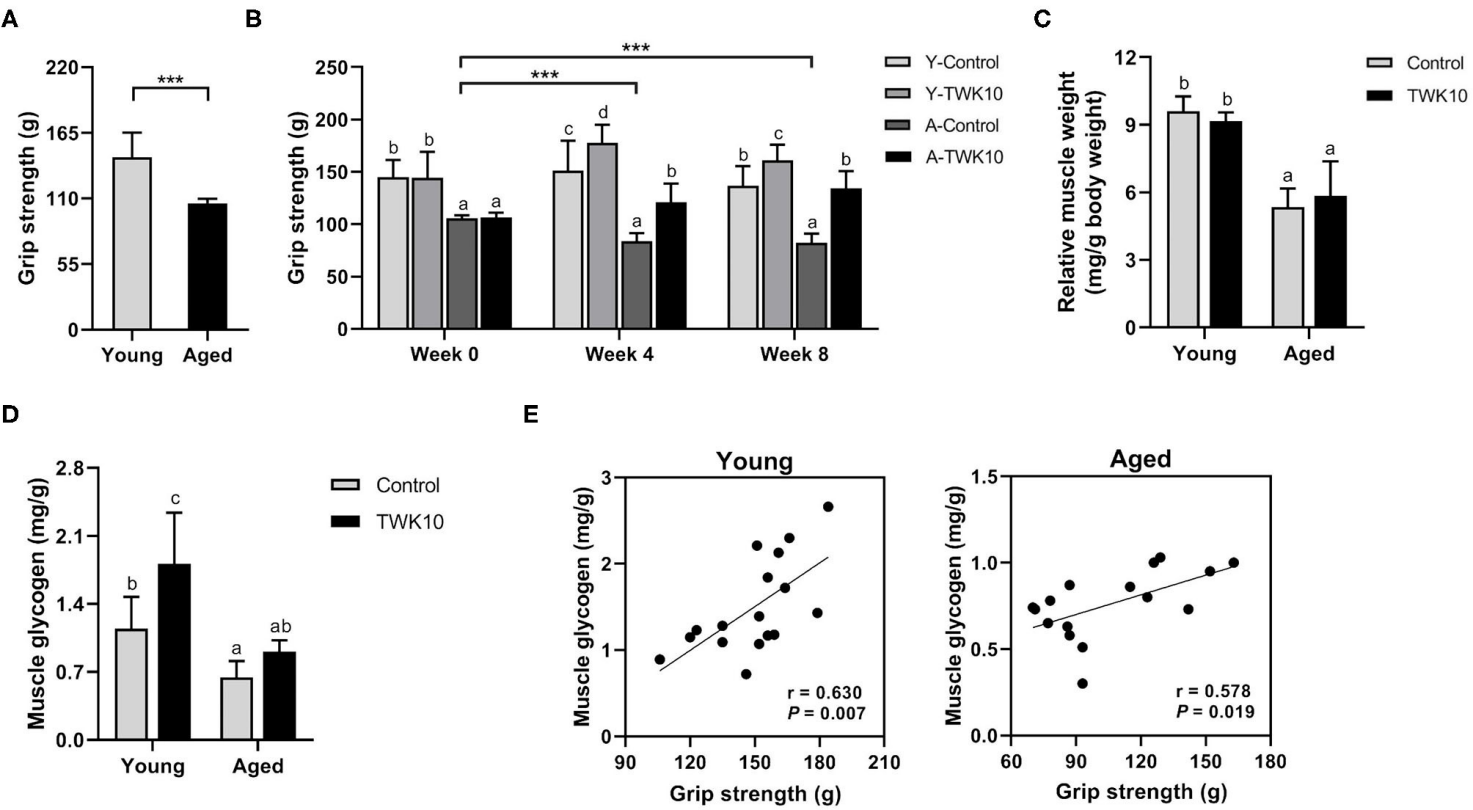
*Lactobacillus plantarum* TWK10 attenuated the age-related decline in skeletal muscle strength by improving muscle quality. **(A)** Forelimb grip strength of young (*n* = 17) and aged mice (*n* = 16) was measured before administration. **(B)** Mice were treated with *Lactobacillus plantarum* TWK10 or PBS for 8 weeks, and forelimb grip strength was measured at week 0, week 4, and week 8. **(C,D)** Gastrocnemius muscles were harvested and subjected to measurements of muscle wet weight and glycogen levels. Data are represented as mean ± SD. Statistical differences among groups were analyzed by two-way ANOVA with the *post-hoc* Tukey-Kramer test. Different letters (a, b, c) indicate significant differences among groups, *P* < 0.05. Two group comparison was analyzed using the unpaired Student *t*-test. ***P < 0.001. **(E)** Pearson's correlation, after 8 weeks of TWK10/PBS administration, between grip strength and muscle glycogen levels in the young and aged mice.

To evaluate baseline levels of muscle strength in the young and aged mouse groups, all mice were subjected to measurements of forelimb grip strength before TWK10 administration. The forelimb grip strength in the aged mouse groups (A-Control and A-TWK10; 106.1 ± 3.7 g, *n* = 16) was significantly (*P* < 0.001) lower than in the young mouse groups (Y-Control and Y-TWK10; 144.7 ± 20.7 g, *n* = 17) (Figure 1A). At week 0, no significant differences in the grip strength between the Y-Control and Y-TWK10 groups, and A-Control and A-TWK10 groups, respectively, while the levels of the grip strength in the young mouse groups were significantly higher (*P* < 0.001) than those in the aged mouse groups. Two-way ANOVA indicated that the factors “aging (young vs. aged)” and “treatment (control vs. TWK10 administration)” significantly (*P* < 0.05) affected the grip strength at week 8. After 4 and 8 weeks of TWK10 administration, the grip strength in the aged mouse groups were significantly lower [Y-TWK10 at week 4 and 8 (*P* < 0.001), and A-TWK10 at week 4 (*P* < 0.001) and week 8 (*P* < 0.01)] than those in the young mouse groups, respectively. Meanwhile, the grip strength in the TWK10 administered groups were significantly higher [Y-TWK10 at week 4 and 8 (*P* < 0.05), and A-TWK10 at week 4 (*P* < 0.05) and week 8 (*P* < 0.01)] compared with those in the Y-Control and A-Control groups, respectively. A significant reduction (*P* < 0.001) in the grip strength was observed in the control aged mice (A-Control) at both weeks 4 and 8, compared with that at week 0 (Figure 1B). Furthermore, the gastrocnemius muscle weights in the aged mouse group were significantly lower (*P* < 0.05), relative to those in the young mouse group. However, no significant differences in the gastrocnemius muscle weights were observed following TWK10 administration in both young and aged mice (Figure 1C). The gastrocnemius muscle glycogen levels were significantly lower in the aged mice (A-Control) compared with young mice (Y-Control). However, on TWK10 administration, the glycogen levels were significantly increased (*P* < 0.05) in the young mouse group, and slightly increased in the aged mice (Figure 1D). Positive correlations between the grip strength and muscle glycogen levels were observed in both young mice (Pearson's correlation coefficient, *r* = 0.630, *P* = 0.007) and aged mice (Pearson's correlation coefficient, *r* = 0.578, *P* = 0.019) (Figure 1E).

### The Effect of TWK10 on Aging-Associated Bone Loss

To evaluate the efficacy of TWK10 in restoring bone quality, murine femur bones were harvested after 8 weeks of treatment, and the cortical and trabecular microstructures were analyzed using micro-CT. The impact of TWK10 on femur BMD and trabecular parameters is summarized in Table 2. Age-related bone loss was demonstrated as a significant reduction (*P* < 0.05) in Tb.N and an increase in Tb.Sp (*P* = 0.1547), when comparing the Y-Control and A-Control groups. The BV/TV values were significantly higher (*P* < 0.05) in the Y-TWK10 group compared to the Y-Control mice, whereas the values of Tb.Sp in the Y-TWK10 group were significantly lower (*P* < 0.05). Administration of TWK10 resulted in a significant increase (*P* < 0.05) in the Tb.N of Y-TWK10 and A-TWK10 mice, compared to the control mice (the Y-Control and A-Control groups). The serum levels of vitamin D in the A-TWK10 were also increased (*P* = 0.0584), compared with those in the A-Control group. No significant differences in Tb.Th, Tb.Pf, SMI, or BMD values were observed among the four groups (Y-Control, Y-TWK10, A-Control, and A-TWK10).

**Table 2:** Bone quality was improved by *Lactobacillus plantarum* TWK10.

	**Group**			
**Item**	**Y-Control**	**Y-TWK10**	**A-Control**	**A-TWK10**
BV/TV (%)	7.61 ± 2.68^a,b^	13.57 ± 5.67^b#1^	4.56 ± 2.56^a^	7.89 ± 10.15^a,b^
Tb.Th (mm)	0.09 ± 0.01^a^	0.09 ± 0.01^a^	0.10 ± 0.01^a^	0.10 ± 0.02^a^
Tb.Sp (mm)	0.53 ± 0.16^a,b^	0.35 ± 0.10^a#2^	0.76 ± 0.25^b^	0.70 ± 0.32^b^
Tb.N (1/mm)	0.92 ± 0.24^b^	1.94 ± 0.43^c^	0.45 ± 0.26^a^	0.91 ± 0.20^b^
Tb.Pf (1/mm)	20.53 ± 3.21^a^	17.40 ± 3.89^a^	19.23 ± 5.40^a^	17.03 ± 4.90^a^
SMI	2.54 ± 0.18^a^	2.32 ± 0.30^a^	2.61 ± 0.34^a^	2.51 ± 0.64^a^
BMD (g/cm^3^)	0.53 ± 0.05^a^	0.53 ± 0.02^a^	0.55 ± 0.04^a^	0.57 ± 0.08^a^
Serum vitamin D (ng/ml)	35.85 ± 6.25^a^	33.99 ± 5.63^a^	30.45 ± 7.89^a^	36.95 ± 2.94^a#3^

*Data are represented as mean ± SD. Statistical differences among groups were analyzed by two-way ANOVA with post-hoc Tukey-Kramer test. Non-parametric data were statistically analyzed by Kruskal-Wallis test with Dunn's test. Within a row, different letters (a,b,c) indicate significant differences between the groups, P < 0.05. Statistical differences between Control and TWK10-adminitstered young groups on BV/TV (P = 0.0274)^#1^ were analyzed by Mann-Whiney U-test. Statistical differences between Control and TWK10-administered young mice groups on Tb.Sp (P = 0.0173)^#2^, and those between Control and TWK10-administered aged mice groups on serum vitamin D (P = 0.0584)^#3^ were analyzed by Student unpaired t-test, respectively. BV/TV, bone volume fraction; Tb.N, trabecular number; Tb.Sp, trabecular spacing; Tb.Pf, trabecular bone pattern factor; BMD, bone mineral density; SMI, structure model index*.

### The Effect of TWK10 on Aging-Related Spatial Learning and Memory Deficits

**Figure 2:**
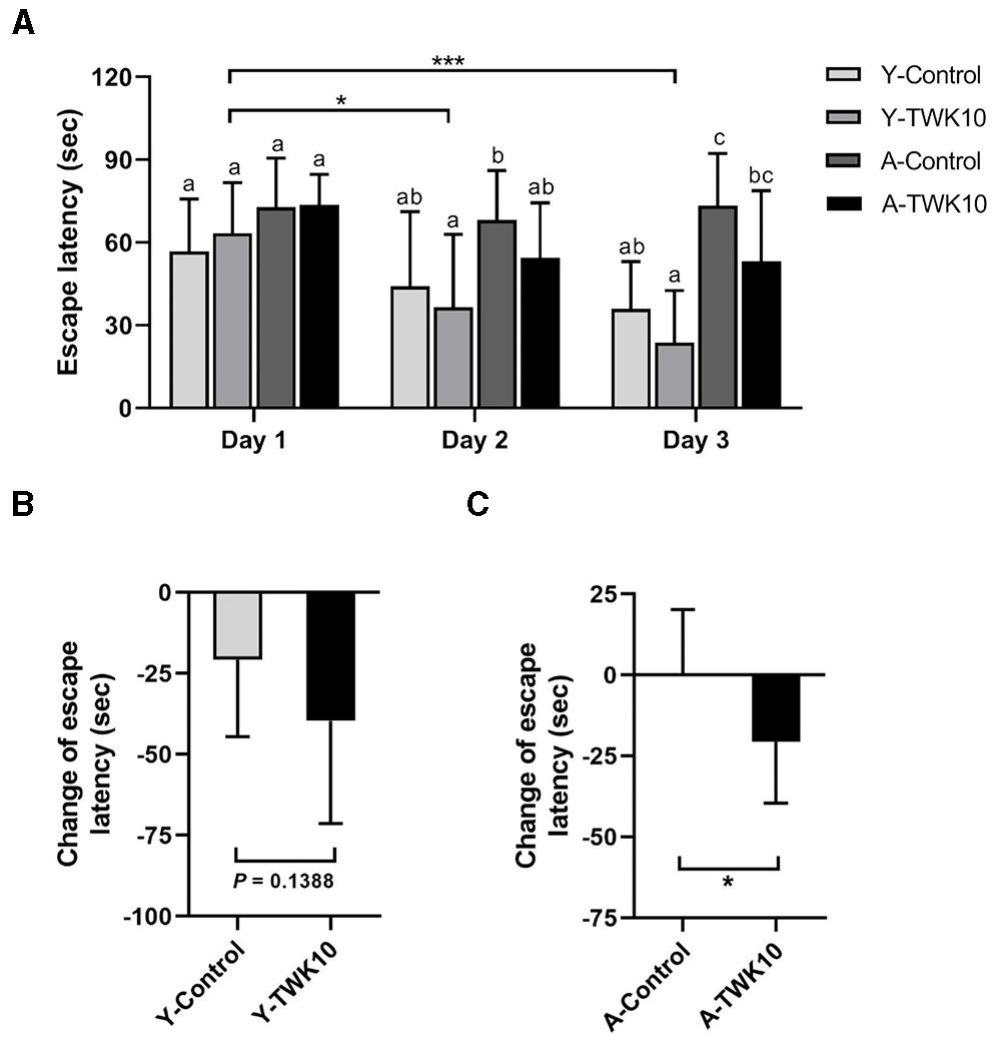
The decline in spatial learning and memory was attenuated by *Lactobacillus plantarum* TWK10. Spatial learning and memory ability were evaluated using the Morris Water Maze after 8 weeks of TWK10 or PBS administration. **(A)** Mean escape latency to the platform were recorded during trial days 1, 2, and 3. **(B,C)** The differences in escape latency between day 1 and day 3 were examined in young and aged mice. Data are represented as mean ± SD. Statistical differences among groups in mean escape latency during the trial days were statistically analyzed by two-way ANOVA with the *post-hoc* Tukey-Kramer test. Different letters (a, b, c) indicate significant differences among the groups, *P* < 0.05. Two group comparison was analyzed using the unpaired Student *t*-test. ***P < 0.001. The differences in delta time of escape latency between the Control and TWK10-administered groups were analyzed using the Mann-Whitney *U*-test. *P < 0.05.

To evaluate the effect of TWK10 on the aging-related decline in spatial learning and memory, the Morris water maze (MWM) test was carried out. Two-way ANOVA indicated that the factor “aging (control vs. aged)” significantly affected (*P* < 0.05) the spatial learning and memory on days 1, 2, and 3, respectively. The mean escape latency of the aged mice (A-Control at day 3) was significantly higher (*P* < 0.01) than that of the young mice (Y-Control at day 3).The mean escape latency of the Y-TWK10 group on day 2 and day 3 was significantly lower (*P* < 0.05, and *P* < 0.01, respectively) compared to that of the Y-TWK10 group on day 1, whereas no significant differences were observed at any of the time points (days 1, 2, and 3) in the Y-Control mice (Figure 2A). The differences in the mean escape latency between day 1 and day 3 in the Y-TWK10 group showed a decreasing trend (*P* = 0.1388) compared to the Y-Control mice (Figure 2B) whereas those in the A-TWK10 group were significantly lower (*P* < 0.05) than those in the A-Control group (Figure 2C).

### The Effect of TWK10 on Body Fat Regulation

**Figure 3:**
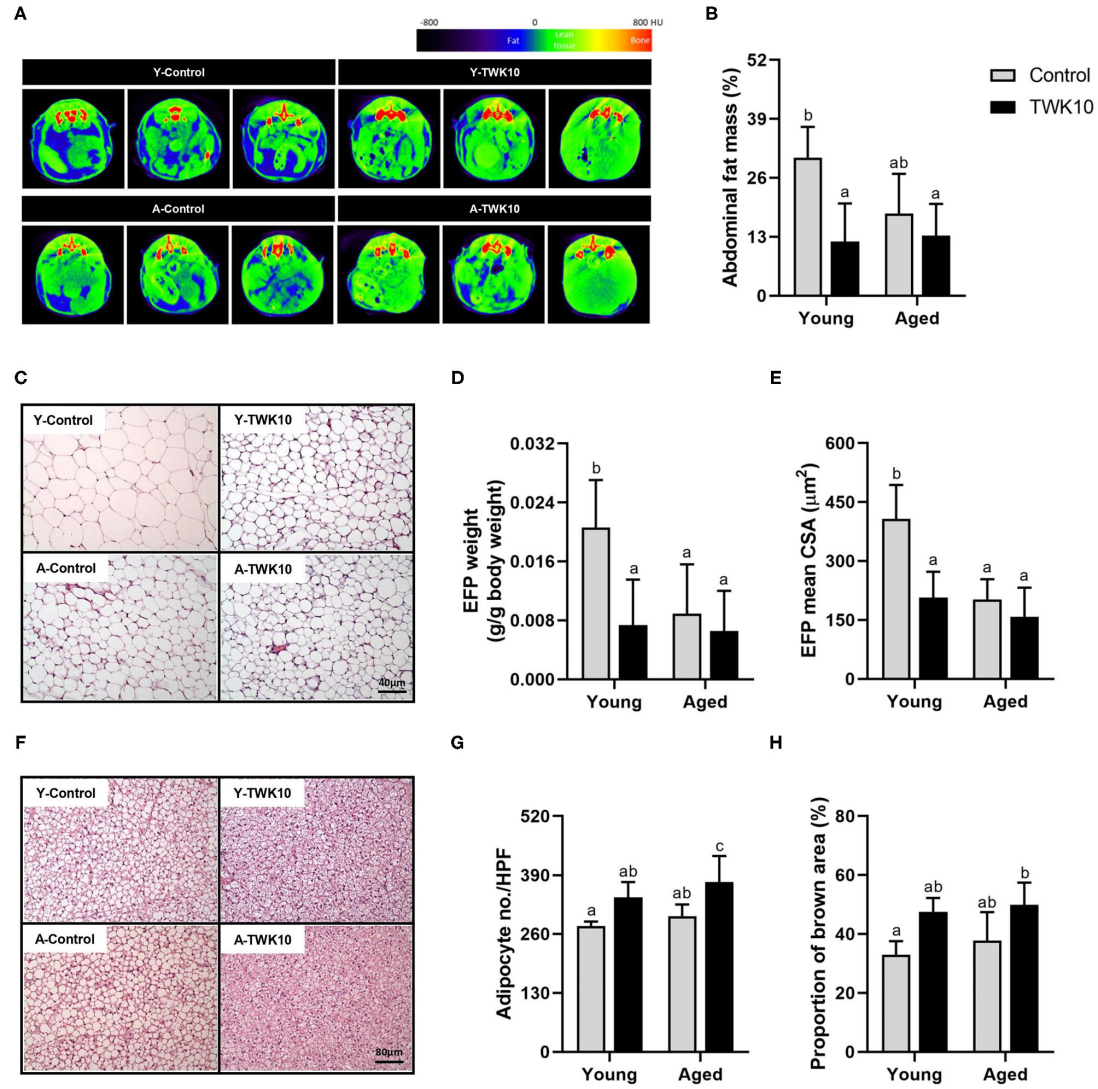
The impact of *Lactobacillus plantarum* TWK10 on body fat regulation. **(A)** Representative micro-CT images showing abdominal region composition. **(B)** Quantification of fat mass at abdominal regions L1 to L5 after 8 weeks of PBS or TWK10 treatment. Epididymal fat pad (EFP) and interscapular brown adipose tissue (BAT) were harvested after 8 weeks of TWK10/PBS administration, and subjected to histological analysis. **(C)** Representative images of EFP obtained by hematoxylin and eosin (H&E) staining. **(D,E)** Measurements of EFP wet weight, and the mean cross-section area of EFP adipocytes. **(F)** Representative images of BAT obtained by H&E staining. **(G)** Quantification of BAT adipocyte number per high-power-field (HPF). **(H)** Quantification of BAT brown area after H&E staining. Data are represented as mean ± SD. Statistical differences among groups were analyzed by two-way ANOVA with the *post-hoc* Tukey-Kramer test and different letters (a, b, c) indicate significant differences, *P* < 0.05. Non-parametric data were statistically analyzed by Kruskal-Wallis test with Dunn's test.

The mean abdominal fat mass (%) of the A-Control group showed a decreasing trend (*P* = 0.1330) when compared to the Y-Control mice. Following the administration of TWK10 for 8 weeks, the mean abdominal fat mass of Y-TWK10 mice became significantly lower (*P* < 0.05) than that of the Y-Control group, whereas no significant differences were observed between the aged mouse groups (Figures 3A,B). Significant interactions (*P* < 0.05) [aging (young vs. aged) × treatment (control vs. TWK10 administration)] were observed in the mean of EFP weights and CSA of adipocytes in EFP, respectively. The mean EFP weight and the mean CSA of adipocytes in EFP of Y-TWK10 mice were significantly lower (*P* < 0.05) than in the Y-Control group (Figures 3C–E). The mean number of BAT adipocytes in the A-Control group was no different compared to the Y-Control mice. However, TWK10 administration resulted in significantly higher BAT adipocyte numbers in A-TWK10 group compared to the control group (Figures 3F,G). To better understand the effect of TWK10 treatment on BAT composition, the relative BAT brown area (%) (visualized following H&E staining), as the proportion of the mitochondria-containing area, was quantified. The mean relative brown area (%) in the BATs of mice in the Y-TWK10 and A-TWK10 groups showed strong increasing trends (*P* = 0.0858) as compared to those in the control groups (Figure 3H).

### The Effect of TWK10 on the Gut Microbiota

To address the impact of TWK10 on the gut microbiota, freshly voided fecal samples were collected following 8 weeks of TWK10/PBS administration and subjected to 16S rRNA gene sequencing analysis. Sequencing of fecal microbiota resulted in a total 5,314,481 quality filtered reads, corresponding to an average of 69,927 reads per sample. After chimera removal, the reads per sample were rarefied to 52,190. Based on the analysis of sequencing data, a total of 580 operational taxonomic units (OTUs) were obtained, with 267 genera of microorganisms identified. Taxonomic and phylogenetic information relating to the OTUs is provided in Supplementary Table 1.

**Figure 4:**
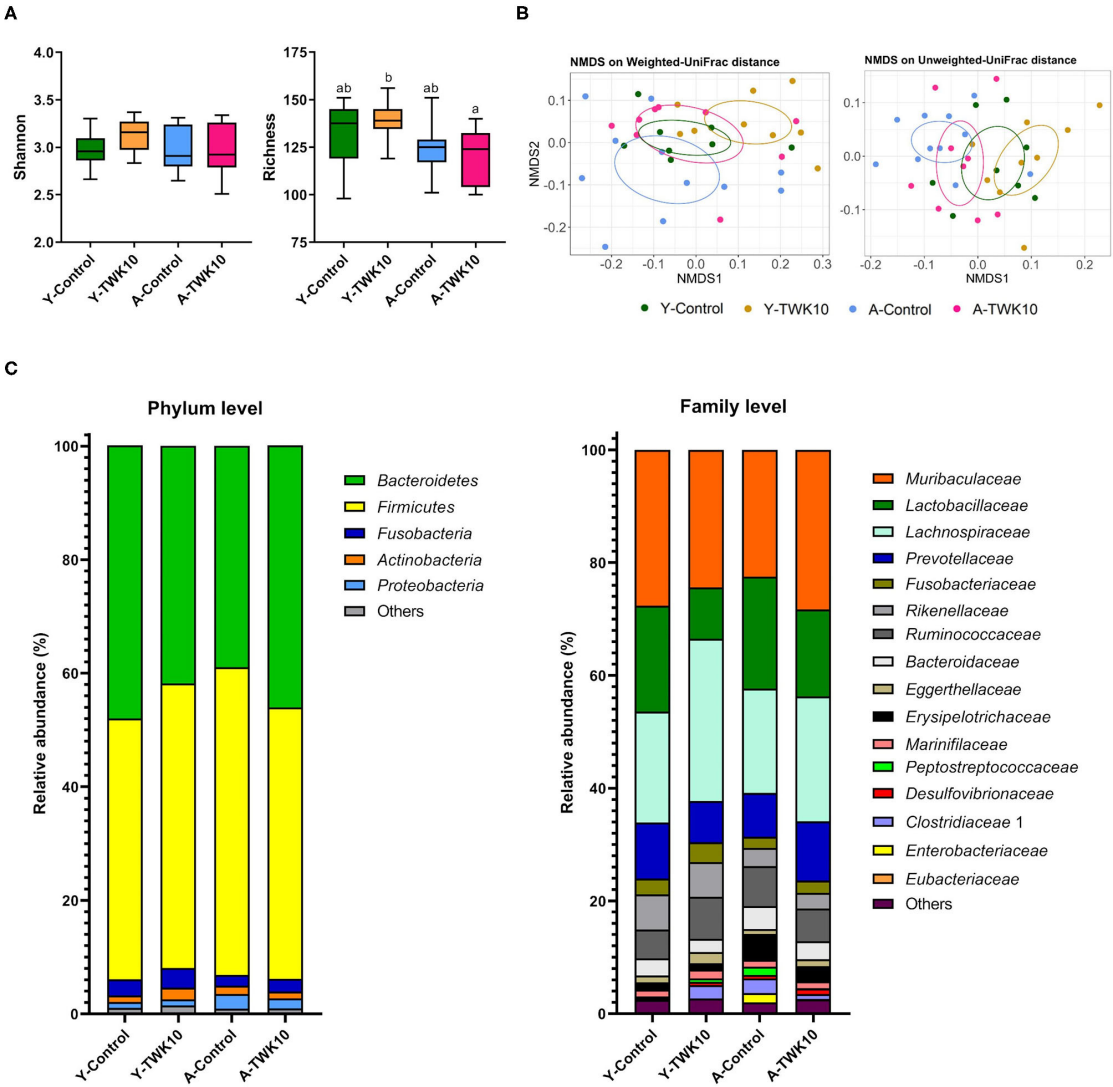
Modulation of gut microbial composition by *Lactobacillus plantarum* TWK10. Gut microbiota signatures were expressed in terms of **(A)** Alpha-diversity (Shannon and Richness indices). The box plots show the smallest and largest values, 25 and 75% quartiles and the median, **(B)** Beta-diversity represented by non-metric multidimensional scaling (NMDS) plots based on weighted and unweighted UniFrac distances, and **(C)** Cumulative bar chart of average relative abundance of bacterial taxa at phylum and family levels. Statistical differences among groups were analyzed by two-way ANOVA with *post-hoc* Tukey-Kramer test, and different letters (a, b, c) indicate significant differences between the groups, *P* < 0.05. Non-parametric data were statistically analyzed by Kruskal-Wallis test with Dunn's test.

To determine how the overall profile of microbial composition was altered by TWK10 administration, alpha-diversity and beta-diversity were analyzed. No significant differences in Shannon indices for α-diversity were observed between the TWK10-treated and control young and aged mice, whereas the richness indices in the A-TWK10 group were significantly lower (*P* < 0.05) than those in the Y-TWK10 group (Figure 4A). To evaluate the differences in the microbial community structures among the four groups, beta-diversities were measured by non-metric multidimensional scaling (NMDS) ordinations of unweighted and weighted UniFrac distance matrices followed by PERMANOVA. As showed in Figure 4B, the fecal microbial composition of the Y-Control group was significantly (PERMANOVA, *P* < 0.05) different from the A-Control group by NMDS analysis with consideration of the presence/absence of taxa (unweighted UniFrac distance). The Y-TWK10 mice showed a distinctly different (PERMANOVA, *P* < 0.05) β-diversity profile compared to the Y-Control mice while considering the presence/absence as well as relative abundance of taxa (weighted UniFrac distance), suggesting that TWK10 induced a rearrangement in the gut microbial composition of young mice. However, the A-TWK10 mice had a similar microbial composition profile to the A-Control group, as determined by NMDS, with weighted UniFrac distance (*P* = 0.309).

To further investigate these alterations in microbial composition, relative abundances of the major taxa at phylum and family levels were analyzed. At the phylum level, the A-TWK10 group showed no significant but slightly higher abundances of *Bacteroidetes* and *Fusobacteria*, with slightly lower abundances of *Firmicutes* and *Proteobacteria* than the A-Control mice. In addition, TWK10 administration tended to decrease the *Firmicutes*/*Bacteroidetes* (F/B) ratio in aged mice (Supplementary Figure 1A). The abundance of *Actinobacteria* in the Y-TWK10 group was significantly higher (*P* < 0.05) than in the Y-Control mice (Figure 4C, Supplementary Figure 1A). At family level, the relative abundances of *Lachnospiraceae* and *Eggerthellaceae* in the Y-TWK10 group were significantly higher (*P* < 0.05) compared to the Y-Control mice, whereas the abundance of *Lactobacillaceae* in the Y-TWK10 group was significantly lower (*P* < 0.01) than in the Y-Control group. The abundances of *Eubacteriaceae* (*P* = 0.0823) and *Ruminococcaceae* (*P* = 0.0927) in the Y-TWK10 group, as well as *Muribaculaceae* (*P* = 0.1119) and *Eggerthellaceae* (*P* = 0.1308) in the A-TWK10 group followed a weak increasing trend toward significance when compared to the abundance of these populations in the Y-Control and A-Control mice, respectively (Supplementary Figure 1B).

To analyze the specific characteristic taxa within each mouse group, LEfSe [Linear discriminant analysis (LDA) Effect Size] was performed based on the discrepancies between groups. Enriched phylotypes in the Y-TWK10 group included the phylum *Actinobacteria* and genera mainly belonging to the class *Clostridia*, namely, *Lachnospiraceae* UCG-006*, Lachnoclostridium, Phascolarctobacterium, Ruminiclostridium* 6*, Tyzzerella*, the *Eubacterium* brachy group, and *Ruminococcaceae* UCG-004, whereas those in Y-Control group belonged to the class *Bacilli* (Supplementary Figure 2A). In A-TWK10 mice, the phylum *Bacteroidetes* was the most differentially abundant bacterial taxon, whereas the microbiota of the A-Control group was dominated by the phylum *Firmicutes* (LDA score [log10] > 3) (Supplementary Figure 2B).

### The Effect of TWK10 on Gut Bacterial Networks

**Figure 5:**
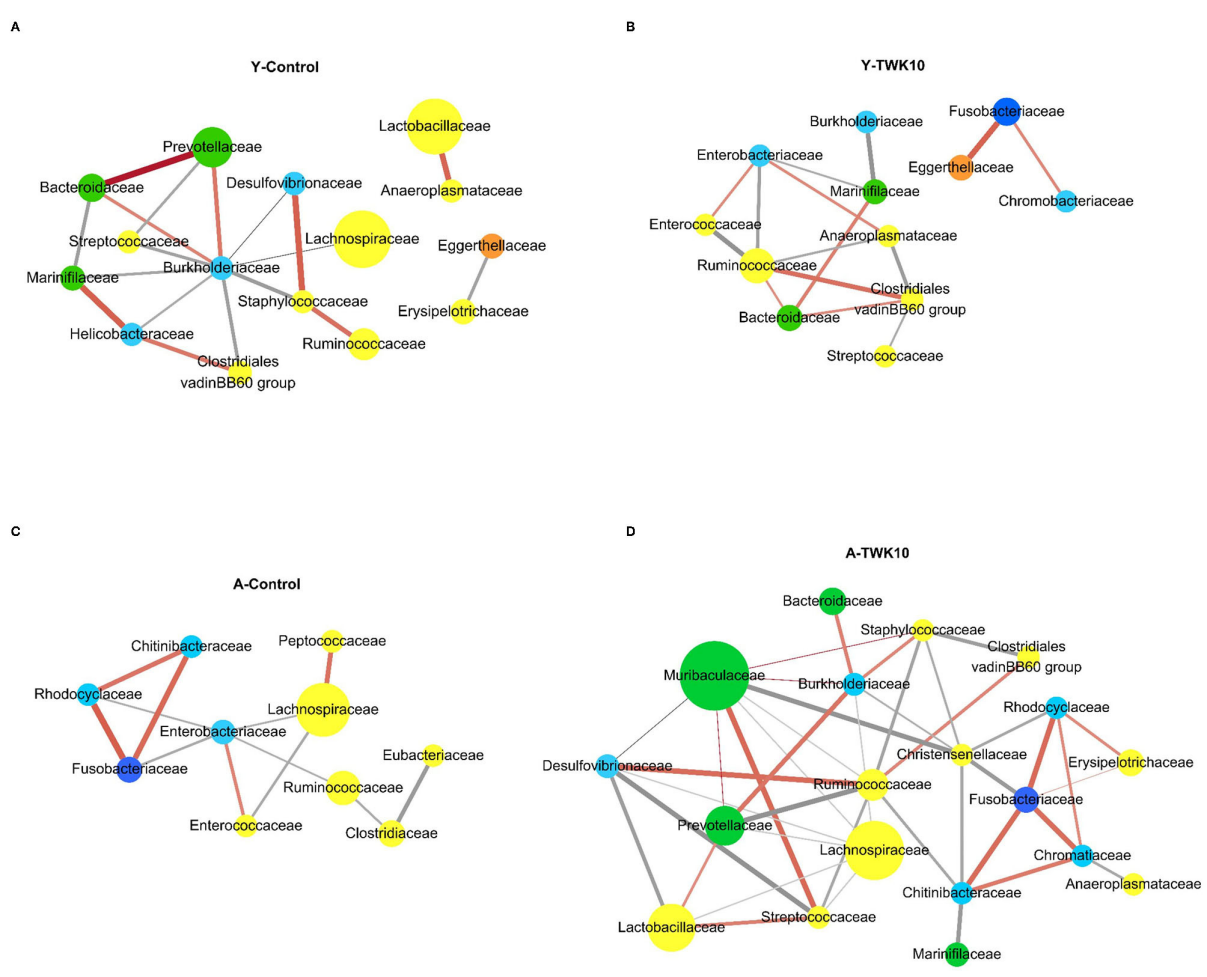
Co-occurrence network analysis of the gut microbiota. Co-occurrence networks were performed at the family level on the basis of relative abundances in the **(A)** young control mice (Y-Control), **(B)** young TWK10-treated mice (Y-TWK10), **(C)** aged control mice (A-Control), and **(D)** aged TWK10-treated mice (A-TWK10). A connection indicated a strong (SParCC's rho cut-off = 0.6) and significant (*P* < 0.01) correlation. Each node represents a family and is colored according to its phylum; yellow: *Firmicutes*, green: *Bacteroidetes*, light blue: *Proteobacteria*, dark blue: *Fusobacteria*, orange: *Actinobacteria*. The node size represents the relative abundance of each family (if >1%) in each group. Each edge represents a positive correlation (orange red line) or a negative correlation (gray line) between the two families with a SparCC's correlation coefficient of ±0.6, and edge shading indicating correlation magnitude.

To investigate the variation in bacterial interactions in the gut microbial community after TWK10 administration in both young and aged mice, microbial correlation network analysis was performed using SparCC. In the Y-Control mice, *Prevotellaceae, Lactobacillaceae*, and *Ruminococcaceae* correlated positively with *Bacteroidaceae* and *Burkholderiaceae, Anaeroplasmataceae*, and *Staphylococcaceae*, respectively (Figure 5A). In the Y-TWK10 group, the predominant *Ruminococcaceae* correlated negatively with *Anaeroplasmataceae, Enterobacteriaceae*, and *Enterococcaceae*, and positively with the *Clostridiales* vadinBB60 group. *Eggerthellaceae* correlated positively with *Fusobacteriaceae* (Figure 5B). In the A-Control group, the most predominant *Lachnospiraceae* correlated negatively with *Enterobacteriaceae* and *Enterococcaceae*, and positively with *Peptococcaceae*. The three families, *Chitinibacteraceae, Fusobacteriaceae*, and *Rhodocyclaceae* showed positive correlations with each other (Figure 5C). In A-TWK10 mice, *Lachnospiraceae* correlated negatively with *Desulfovibrionaceae, Lactobacillaceae, Muribaculaceae, Prevotellaceae, Ruminococcaceae*, and *Streptococcaceae*. *Ruminococcaceae* correlated negatively with *Chitinibacteraceae, Prevotellaceae, Staphylococcaceae, Streptococcaceae*, and *Lachnospiraceae*, and positively with the *Clostridiales* vadinBB60 group and *Desulfovibrionaceae*. *Muribaculaceae* correlated positively with *Streptococcaceae*, and negatively with *Christensenellaceae*. The four families, *Chitinibacteraceae, Chromatiaceae, Fusobacteriaceae*, and *Rhodocyclaceae* showed positive correlations with each other. Three of them (*Chitinibacteraceae, Fusobacteriaceae*, and *Rhodocyclaceae*) also correlated negatively with *Christensenellaceae* (Figure 5D).

### The Effect of TWK10 on the Abundance of SCFA-Producing Gut Bacteria and Gut SCFA Levels

Short-chain fatty acids (SCFAs), such as acetate, propionate, and butyrate are important metabolites in the maintenance of intestinal homeostasis. We therefore next investigated whether TWK10 could facilitate the production of SCFAs through the modulation of gut microbiota in young and aged mice. The concentrations of acetate, propionate, as well as butyrate in the cecal contents of Y-TWK10 mice were significantly (*P* < 0.05) higher, compared to those in the Y-Control group, whereas no significant differences were observed between the A-Control and A-TWK10 groups.

In young mice, we found that the administration of TWK10 resulted in higher abundances of SCFA-producing bacteria. In the case of acetate-producing bacteria, members of the family *Peptococcaceae* (*P* < 0.01) and genus *Ruminococcaceae* UCG-004 (*P* < 0.01) were significantly higher in abundance, with strong increasing trends being observed for the genera *Prevotella* 9 (*P* = 0.1111), *Ruminiclostridium* 6 (*P* = 0.0755), and *Ruminiclostridium* 9 (*P* = 0.0745), in Y-TWK10 mice compared to those in the Y-Control group. The abundances of butyrate-producing bacteria, namely members of the family *Lachnospiraceae* (*P* < 0.05) and the genus *Lachnospiraceae* UCG-006 (*P* < 0.001) were significantly increased as a result of TWK10 administration, and significant increasing trends were observed for members of the family *Ruminococcaceae* (*P* = 0.0927) and the genera *Eubacterium* spp. (*P* = 0.1388) and *Roseburia* (*P* = 0.1388) in Y-TWK10 mice, compared to those in the Y-Control group. Administration of TWK10 to aged mice increased the abundance of acetate-producing bacteria, particularly belonging to the genus *Prevotellaceae* UCG-001 albeit not significantly (*P* = 0.1519). There were no significant differences in the abundances of butyrate-producing bacteria in the A-TWK10 group compared to the control mice. In addition, the propionate-producing genus *Phascolarctobacterium* was observed in both Y-TWK10 and A-TWK10 groups (Table 3).

**Table 3:** *Lactobacillus plantarum* TWK10 enriched SCFA-producing gut bacteria and promoted the production of SCFAs.

	**Group**					
	**Y-Control**	**Y-TWK10**	***P-*value**	**A-Control**	**A-TWK10**	***P*-value**
**SCFA (μmol/g cecal content)**						
Acetate	28.01 ± 26.91	119.90 ± 101.20	0.0130	44.00 ± 26.70	49.08 ± 45.08	0.7823
Butyrate	10.55 ± 7.86	46.04 ± 43.53	0.0211	15.47 ± 12.15	23.13 ± 28.11	0.4724
Propionate	3.50 ± 3.06	12.18 ± 10.34	0.0212	6.57 ± 3.85	6.64 ± 6.56	0.9802
**Acetate-producing bacteria (%)**						
*Bacteroides*	3.0305 ± 2.3119	2.2954 ± 1.5848	0.8148	4.0741 ± 2.7387	3.2048 ± 1.7169	0.5027
*Blautia*	0.0957 ± 0.0392	0.1562 ± 0.0951	0.2766	0.4425 ± 0.4702	0.5243 ± 0.8987	0.8868
*Eggerthellaceae*	1.1709 ± 0.5187	2.0119 ± 1.1364	0.0360	0.9072 ± 0.3313	1.2209 ± 0.4666	0.1308
*Fusobacteriaceae*	2.8120 ± 0.7184	3.4779 ± 1.3763	0.4234	1.9417 ± 0.8716	2.1964 ± 1.0041	0.6556
*Prevotellaceae* UCG-001	6.6855 ± 4.3685	5.6362 ± 2.8759	0.7430	6.0641 ± 3.4124	9.1604 ± 4.1470	0.1519
*Prevotella* 9	0.0081 ± 0.0044	0.0107 ± 0.0042	0.1111	0.0083 ± 0.0020	0.0059 ± 0.0018	0.4000
*Peptococcaceae*	0.0977 ± 0.0594	0.2516 ± 0.1093	0.0016	0.1171 ± 0.0777	0.1707 ± 0.0933	0.2060
*Ruminococcaceae* UCG-004	0.0390 ± 0.0255	0.1144 ± 0.0631	0.0016	0.0677 ± 0.0623	0.0759 ± 0.0673	0.8238
*Ruminiclostridium* 9	0.2943 ± 0.1438	0.5067 ± 0.2774	0.0745	0.2439 ± 0.2088	0.4522 ± 0.4725	0.4119
*Ruminiclostridium* 6	0.0269 ± 0.0385	0.1137 ± 0.1328	0.0755	0.0989 ± 0.0983	0.2440 ± 0.2440	0.4396
*Streptococcus*	0.1604 ± 0.1029	0.2083 ± 0.1344	0.5414	0.0831 ± 0.0626	0.1167 ± 0.0836	0.3562
**Propionate-producing bacteria (%)**						
*Megamonas*	0.0060 ± 0.0017	0.0068 ± 0.0012	0.8000	0.0055 ± 0.0029	0.0047 ± 0.0010	0.8000
*Phascolarctobacterium*	n.d.	0.0061 ± 0.0021	–	n.d.	0.0040 ± 0.0000	–
**Butyrate-producing bacteria (%)**						
*Eubacterium* spp.	0.2803 ± 0.3138	0.5180 ± 0.2924	0.1388	0.4041 ± 0.4617	0.5456 ± 0.8300	0.7103
*Faecalibacterium prausnitzii*	0.0086 ± 0.0022	0.0141 ± 0.0019	0.2000	0.0107 ± 0.0051	0.0075 ± 0.0000	–
*Lachnospiraceae*	19.6801 ± 7.6001	28.7954 ± 9.6799	0.0464	18.4889 ± 12.9901	22.1170 ± 13.0379	0.4561
*Lachnospiraceae* UCG-006	0.1681 ± 0.1416	1.9809 ± 2.0960	<0.001	1.3084 ± 2.3146	0.4179 ± 0.2934	0.9048
*Odoribacter*	1.1620 ± 0.6283	1.5569 ± 0.9212	0.3704	1.2450 ± 0.6954	1.2187 ± 0.8926	0.8820
*Oscillibacter*	0.4131 ± 0.3367	0.5855 ± 0.5635	0.6058	0.3599 ± 0.3216	0.4936 ± 0.4787	0.8238
*Roseburia*	0.1446 ± 0.1095	0.3088 ± 0.2865	0.1388	0.3245 ± 0.2431	0.4554 ± 0.6009	> 0.9999
*Ruminococcaceae*	5.1385 ± 2.0922	7.4884 ± 2.7297	0.0927	7.0488 ± 4.4442	5.7306 ± 2.9812	0.6027

*Relative abundances of SCFA-associated gut bacteria and the contents of cecal SCFAs in young and aged mice. Data are represented as mean ± SD. Statistical differences between Control and TWK10-administered groups on the basis of the relative abundances of SCFA-producing gut bacteria were analyzed by Mann-Whiney U-test. Statistical significances between the cecal SCFA content of Control and TWK10-administered groups were analyzed by Student unpaired t-test. P < 0.05 represents statistically significant differences. n.d, not detected*.

### The Correlation Between Gut Microbiome Composition and TWK10-Mediated Health Benefits

**Figure 6:**
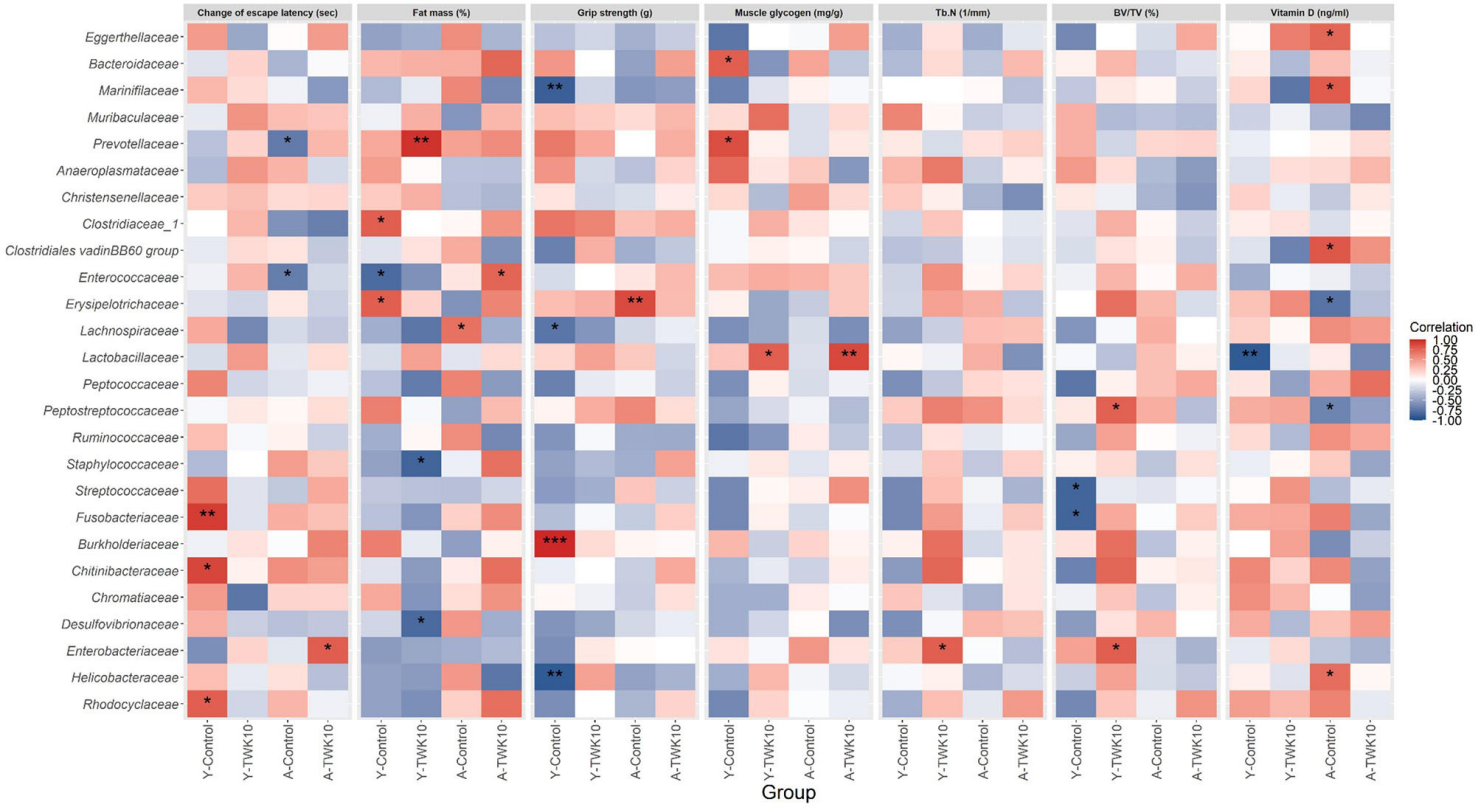
Heatmap of Spearman's correlation analysis between the gut microbiota and the altered age-related host phenotypic features. Spearman's correlation analysis was performed to investigate the correlations between the relative abundances of 26 major families and the values of seven altered aged-related host phenotypic features selected from the differential analysis between Y-Control and Y-TWK10 or A-Control and A-TWK10 groups. Red squares indicate positive correlations and blue squares indicate negative correlations. *P < 0.05, **P < 0.01, ***P < 0.001.

The correlation between the relative abundance of gut bacterial taxa and seven age-related host phenotypic features: including spatial learning and memory, muscle and bone qualities, in young and aged mice, were assessed using Spearman's correlation analysis. The escape latency was significantly lower following the treatment of aged mice with TWK10 (A-TWK10 group, Figure 2C). The change in escape latency was significantly and positively correlated with the abundance of the family *Enterobacteriaceae* (Figure 6), whereas the abundance of *Enterococcaceae* and *Prevotellaceae* populations in A-Control mice were negatively correlated with the change in escape latency. In young mice, the abundance of *Chitinibacteriaceae, Fusobacteriaceae*, and *Rhodocyclaceae* (in the Y-Control group) correlated positively with the change in escape latency. The reduction in abdominal fat mass (%) mediated by TWK10 administration to Y-TWK10 mice was significantly positively and negatively correlated with the presence of *Prevotellaceae*, and *Desulfovibrionaceae* and *Staphylococcaceae* populations, respectively. Meanwhile, *Enterococcaceae* abundance correlated positively with the fat mass of A-TWK10 mice. The abundance of *Clostridiaceae* and *Erysipelotrichaceae*, and *Enterococcaceae* were positively and negatively correlated with the fat mass in the Y-Control group, respectively, while *Lachnospiraceae* abundances correlated positively with the fat mass of A-Control mice. For muscle aging-associated parameters, *Burkholderiaceae* abundances correlated positively with grip strength, whereas *Helicobacteraceae, Lachnospiraceae*, and *Marinifilaceae* populations correlated negatively with grip strength, in the Y-Control group. In aged mice, the presence of *Erysipelotrichaceae* correlated positively with grip strength in the A-Control group, whereas no significant correlations were observed in the A-TWK10 or Y-TWK10 mice. Additionally, muscle glycogen levels were increased by TWK10 administration in both young and aged mice (Figure 1D), and correlated positively with the abundance of *Lactobacillaceae*. In the Y-Control group, the abundance of *Bacteroidaceae* and *Prevotellaceae* correlated positively with muscle glycogen levels, whereas no significant correlations were observed in the A-Control group (Figure 6). Strong negative correlations between phylum *Proteobacteria* abundance and levels of muscle glycogen in the A-TWK10 mice were also observed (Supplementary Figure 3). In relation to bone health parameters, Tb.N and BV/TV values were higher in young and aged groups after TWK10 administration (Table 2). *Enterobacteriaceae* correlated positively with Tb.N in the femur of Y-TWK10 mice. There were no bacterial families showing strong correlations with Tb.N in the aged mouse groups. In the Y-TWK10 mice, however, *Enterobacteriaceae* and *Peptostreptococcaceae* presence correlated positively with BV/TV, whereas *Fusobacteriaceae* and *Streptococcaceae* populations correlated negatively with BV/TV in the Y-Control group. Serum vitamin D levels in A-TWK10 were also higher when compared to the A-Control mice (*P* = 0.0584) (Table 2). However, no significant correlations between bacterial taxa and serum vitamin D levels were observed in the A-TWK10 group. The abundance of the *Clostridiales* vadinBB60 group members as well as *Eggerthellaceae, Helicobacteraceae*, and *Marinifilaceae* correlated positively with serum vitamin D levels in A-Control mice, whereas *Erysipelotrichaceae* and *Peptostreptococcaceae* exhibited a negative correlation. In the Y-Control mice, *Lactobacillaceae* abundance correlated negatively with serum vitamin D levels.

## Discussion

### The Effect of TWK10 on Muscle Strength

Aging usually brings about anabolic impairment of skeletal muscle, which in turn leads to reductions in muscle mass and strength (45). Mounting evidence has indicated that declining muscle mass and strength is closely associated with mortality rates in the elderly. Of the two, it appears that muscle strength is more important than muscle mass as a determinant of functional limitation and poor health in the elderly (2, 3, 46–49). Glycogen is an essential energy substrate supporting skeletal muscle activity in humans and animals, and the rate of glycogenesis in muscle decreases with age (50). As demonstrated in our previous studies, TWK10 administration enhanced muscle strength and increased muscle mass, and improved muscle quality and endurance performance in mice and humans (30, 31). However, the impact of TWK10 on slowing down the progression of muscle aging was not clear.

In the present study, we aimed to demonstrate the effect of oral TWK10 administration on the progression of aging-related disorders, such as muscle weakness in naturally aging mice. Therefore, the forelimb grip strength (a marker used widely for the evaluation of *in vivo* neuromuscular performance), gastrocnemius muscle weight, and muscle glycogen levels were determined in young and aged mice.

As our results showed, a significant reduction in the forelimb grip strength (*P* < 0.001), muscle weight (*P* < 0.05), and glycogen levels (*P* < 0.05) was observed in aged mice when compared to young mice (Figures 1A–D). Although TWK10 administration did not seem to significantly increase the muscle weight of either young or aged mice when compared to those in the control groups, it did significantly increase their grip strength and muscle glycogen levels. In addition, significant positive correlations between the grip strength and muscle glycogen levels were observed in both the young (*P* < 0.01) and aged (*P* < 0.05) mice (Figure 1E). These findings indicate that the muscle strength-improving benefits exerted by TWK10 could be attributed to the improvement in muscle quality, specifically by increasing glycogen concentration in the muscle tissue. The effect of TWK10 on improving muscle strength and preventing muscle loss in aged mice is consistent with a previous study investigating the use of *L*. *paracasei* PS23 in senescence-accelerated mouse prone-8 (SAMP8) mice (27). In our previous study, we found that TWK10 administration could boost muscle mass in 6-week-old mice (30). However, in the present study, muscle mass was not increased by TWK10 administration (Figure 1C), although the myofiber cross-sectional area was enlarged in TWK10-treated young mice (data not shown; Y-Control vs. Y-TWK10 groups, 531 ± 73 vs. 641 ± 92 μm^2^). These inconsistent results relating to muscle mass may be due to the difference in the ages of study animals. Generally, 3–6 month-old mice are considered as mature adults, and are usually used as the reference group in aging-associated studies. Since mice undergo a period of rapid growth until they reach 2–3 months of age, administration of TWK10 during the developmental stage may promote growth more easily than at the mature adult stage.

Muscular glycogen content affects muscle quantity and muscle quality, together with mitochondrial quality, dietary nutrition, and the levels of inflammation-related cytokines and anabolic hormones. Although the above results confirmed that TWK10 administration significantly improved muscle strength in aged mice, further investigations are needed to elucidate the underlying mechanisms involved. In addition, Khandelwal et al. (51) reported that the reduced activities of liver glycogen synthase and phosphorylase in aged rats are indicative of a likely diminution in the turnover of glycogen in liver during aging. The synthesis of glycogen in muscle decreases with age (50). Skeletal muscle is the major tissue where insulin stimulates glucose uptake from the blood via translocation of GLUT4 (52). Therefore, TWK10 may play a role in regulation of age-associated deficit in glycogen metabolism contributing to the generally observed glucose intolerance upon aging.

### The Effect of TWK10 on Bone Quality

Osteoporosis is a common age-related disorder, which is characterized by reduced bone mass and density in the aged population (6). With aging or the onset of osteoporosis, BV/TV, Tb.Th, Tb.N, and BMD decrease, whereas Tb.Sp, Tb.Pf, and SMI usually increase (53). Vitamin D is an essential factor involved in calcium metabolism and is therefore necessary for bone formation. With aging, the risk of vitamin D deficiency is also significantly increased (54). In the A-Control mice, aging-related bone loss was observed by detecting a significant reduction in Tb.N (*P* < 0.05), accompanied by a slight increase in Tb.Sp (*P* = 0.1547), when compared to the Y-Control group. TWK10 administration significant increased (*P* < 0.05) the BV/TV and Tb.N values in both young and aged mice, while significantly decreasing (*P* < 0.05) Tb.Sp in young mice. A near-significant increase (*P* = 0.0584) in serum vitamin D levels was also observed in A-TWK10 mice (Table 2). These results demonstrate that TWK10 administration not only showed a protective potential against age-related bone loss but also improved bone quality in mice, which is consistent with a previous study investigating the effect of *L*. *paracasei* GKS6 and *L*. *plantarum* KM3 supplementation in ovariectomized SAMP8 mice (29).

### The Effect of TWK10 on Body Fat

In general, aging not only brings about the loss of lean mass but increases the amount of body fat (55). With aging, fat is redistributed from the subcutaneous to the visceral region. Accumulation of adipose tissue in the abdominal compartment is associated with an increased risk of chronic disease, such as cardiovascular disease, insulin resistance, and type 2 diabetes mellitus (56, 57). Here, we evaluated the age-associated alterations in body composition and the impact of TWK10 on body fat by micro-CT. We found that TWK10 administration significantly lowered (*P* < 0.05) the mean abdominal fat mass, mean EFP weight, and mean CSA of EFP adipocytes in young mice compared to the Y-Control group (Figures 3A–E), which was consistent with our previous findings (30). Although we expected to see the age-associated increase in body fat in the A-Control group (compared to the Y-control mice), no significant change in fat mass was observed. As Hamrick et al. (58) have previously reported, this unfavorable result may be due to the significant decline in fat mass that occurs naturally in aging mice. Since the aged mice used in this study were 27–30 months old at the time of fat mass evaluation, they may have been too old to investigate the impact of TWK10 on the age-associated accumulation in body fat. In addition, aging is associated with the reduction in BAT mass and activity (59). The reduction in BAT that occurs with aging is linked to the loss of mitochondrial function, increased inflammation, impairment of the sympathetic nervous system, and changes in endocrine signaling (60–63). According to our data, no significant changes affecting BAT were detected when comparing the Y-Control and A-Control mice. However, TWK10 administration increased the mean adipocyte number and the size of the area containing mitochondria in both the Y-TWK10 and A-TWK10 mice (Figures 3F–H), suggested that TWK10 may facilitate the browning of adipose tissue. To the best of our knowledge, BAT is crucial for heat generation and energy consumption. It is thus worthwhile to further evaluate BAT activity and the brown-to-white fat ratio in order to elucidate the impact of TWK10 on body fat. These results demonstrate that TWK10 has the potential to alter body composition, promoting a healthier configuration and improving metabolism.

### The Effect of TWK10 on Learning and Memory

Impairment of learning and memory is another common health issue affecting the elderly. The Morris water maze (MWM), which assesses rodent spatial learning and memory, is one of the most widely used behavioral tests (35). We conducted the MWM test to evaluate the effect of TWK10 on aging-related spatial learning and memory decline in mice.

We observed a significant increase (*P* < 0.001) in escape latency with aging, whereas TWK10 administration significantly lowered escape latency on days 2 and 3 in young mice (Figure 2A). The change in escape latency between days 1 and 3 for the A-TWK10 and Y-TWK10 mice showed a significant decrease (*P* < 0.05) and a decreasing trend (*P* = 0.1388), respectively (Figures 2B,C). These results demonstrate that TWK10 improved the learning and memory capacities of young mice, and attenuated memory loss in aged mice. These observations are consistent with previous studies: *L*. *paracasei* PS23 in SAMP8 mice (26), probiotic mixture of *L. paracasei* BCRC 12188, *L. plantarum* BCRC 12251 and *Streptococcus thermophilus* BCRC 13869 in D-galactose-induced aging C57BL/6 mice (64), and a multi-strain probiotic preparation composed of *Bifidobacterium bifidum, Bifidobacterium lactis, L*. *acidophilus*, and *Lactobacillus casei* in SAMP8 mice (65).

### The Effect of TWK10 on the Gut Microbiota

Age-related degeneration is usually associated with gut microbiota imbalance (9, 11, 66–68). Our results are in agreement with previous findings showing that aging alters the gut microbiota in mice (69). The fecal microbial structures of A-Control mice was enriched in *Firmicutes*, with a lower abundance of *Bacteroidetes*, compared to the Y-Control group (Supplementary Figure 1A). Larger *Firmicutes* and smaller *Bacteroidetes* populations are commonly associated with a dysbiotic microbial signature and poor health (70, 71). Probiotics modulate gut microbial composition imbalances and confer beneficial functions to gut microbial communities (72). Our results indicate that TWK10 administration reduced the *Firmicutes*/*Bacteroidetes* (F/B) ratio in aging mice compared to the A-Control group (Supplementary Figure 1A), although this decrease was not statistically significant, which could be due to the small sample size used in our study. This finding suggests that TWK10 administration could modify the F/B ratio to restore gut microbial balance and confer health benefits.

Higher abundances of phylum *Proteobacteria* and family *Enterobacteriaceae* members were present in aged mice compared to young mice (Supplementary Figures 1A,B). The phylum *Proteobacteria* comprises a wide variety of Gram-negative pathogens, including the families *Enterobacteriaceae, Pseudomonadaceae, Vibrionaceae*, and *Yersiniaceae*. Among them, *Enterobacteriaceae* represents a core bacterial group, comprising over 30 genera and 130 species. These enterobacteria include potentially pathogenic bacteria (termed pathobionts), which exert specific effects on the host mucosal immune system and are the primary cause of infections when host resistance mechanisms fail as a result of aging (73, 74). In addition, the aging-related increase in the intestinal abundance of *Enterobacteriaceae* and other Gram-negative bacteria may result in an increased endotoxin challenge against the weakened intestinal barrier, leading to chronic inflammation (74). Our results show that TWK10 was able to reduce the abundance of *Enterobacteriaceae* in aged mice (Supplementary Figure 1B). Significant reductions in the abundances of major families in the phylum *Proteobacteria*, such as *Chitinibacteraceae, Enterobacteriaceae, Helicobacteraceae, Rhodobacteraceae*, and *Rhodocyclaceae* (except *Burkholderiaceae*) in young and aged mice after TWK10 administration were observed (Supplementary Figure 4). These findings are consistent with previous reports (75, 76), which suggested that TWK10 has the potential to promote gut health in the elderly.

Furthermore, we found differences in the microbial community co-occurrence patterns between young and aged mice. The co-occurrence networks indicated that microorganisms belonging to the phylum *Bacteroidetes* had strong positive correlations in the Y-Control group (Figure 5A). Meanwhile in the A-Control mice, Gram-negative opportunistic pathogens belonging to the phylum *Proteobacteria* (*Chitinibacteraceae, Enterobacteriaceae*, and *Rhodocyclaceae*), and the family *Enterococcaceae* were positively correlated with each other (Figure 5C). In the Y-TWK10 group, the topology of the co-occurrence network was altered after TWK10 administration. As the most abundant bacterial families in the main network of the Y-TWK10 group, *Ruminococcaceae* showed mutual restriction with *Anaeroplasmataceae, Enterobacteriaceae*, and *Enterococcaceae* (Figure 5B). The family *Christensenellaceae*, which is linked to metabolic health and longevity (77–80), showed negative correlations with *Chitinibacteraceae, Fusobacteriaceae*, and *Rhodocyclaceae*. Additionally, we also found that the connections between pathogenic microorganisms were disrupted by TWK10 administration in aged mice (Figure 5D), which suggested that the disruption of the correlated interactions among pathogenic microorganisms may have potential applications in slowing the aging process.

Gut microbiota-derived SCFAs, including acetate, propionate, and butyrate, are key mediators in the maintenance of gut and metabolic health (20, 81, 82). An increase in microbial SCFA production is regarded as beneficial for health. Lactate, which is produced by lactic acid bacteria (LAB) as the major end-product of sugar fermentation, is also one of the important growth factors for SCFA-producing gut bacteria (83). Therefore, LAB are extensively used as probiotics. (84, 85). Chen et al. (86) reported that the administration of *L. paracasei* PS23 significantly decreased the abundance of butyrate-producing *Lachnospiraceae* UCG 001 in aged SAMP8 mice. In contrast, we observed that on TWK10 administration, the abundance of *Lachnospiraceae* was significantly increased (*P* = 0.0464) in Y-TWK10 mice and maintained at a high level (comprising > 20% of total fecal microbiota) in the A-TWK10 group. Wang et al. (87) reported that probiotic *L*. *plantarum* P-8 could improve human gastrointestinal health by increasing the fecal concentrations of acetate and propionate in all age groups, including the elderly. In addition, probiotic *L. acidophilus* DDS-1 modulated intestinal microbiota by increasing *Akkermansia muciniphila* and *Lactobacillus* spp. and reducing *Proteobacteria* spp. abundances, which was accompanied by an increase in cecal propionate and butyrate levels in aged C57BL/6J mice (21, 22). Given that TWK10 administration appears to modulate the gut microbiota of young and aged mice, we hypothesized that TWK10 could be used to boost gut SCFA production. Consequently, we found no significant difference in the levels of cecal SCFAs between the Y-Control and A-Control groups, whereas a significant increase in SCFA levels was observed in the Y-TWK10 group compared to Y-Control mice (Table 3). An overall increase in acetate- and butyrate-producing bacteria was observed in the Y-TWK10 group compared to Y-Control mice. We also observed that TWK10 administration caused a slight increase in the gut acetate and butyrate levels of aged mice. These results imply that TWK10 may exert its health benefits via microbiota modulation and consequently SCFA production, especially in young mice.

In our previous study, we have demonstrated that TWK10 administration significantly increased skeletal muscle mass and strength in mice (30). Growing evidence has led to the notion of the ‘gut-muscle axis', which implies that gut microbiota may act as the mediator for muscle health (88, 89), and hold therapeutic promise for muscle-related diseases, such as sarcopenia (90). Numerous studies have highlighted the close relationship between muscle glycogen levels and fatigue resistance (91). However, the mechanism linking muscle glycogen concentration to muscle function remains elusive. SCFAs have been shown to increase skeletal muscle glycogen levels (92–95), and have the potential to increase skeletal muscle mass and physical function (96). In the present study, we found that the muscle glycogen levels were significantly and positively correlated with muscle strength in young (*P* = 0.007) and aged (*P* = 0.019) mice (Figure 1E), and the abundances of *Lactobacillaceae* members displayed significant positive correlations with muscle glycogen levels in Y-TWK10 and A-TWK10 mice (Figure 6). We also evaluated whether altered age-related host phenotypic features in each treatment group correlated with the SCFA levels. However, only a few of these correlations were statistically significant (*P* < 0.05). In the young mouse group, muscle glycogen concentration was positively correlated with SCFA levels after TWK10 administration. In addition, a negative correlation between butyric acid and muscle glycogen levels was observed in the Y-Control group (Supplementary Figure 5). It remains important to determine whether these correlations arose as a consequence of TWK10 administration. Further studies are needed to understand the mechanisms by which SCFAs produced by gut microbiota affect skeletal muscle following TWK10 administration.

## Conclusions

In the present study, we have for the first time presented evidence of TWK10 attenuating aging-related disorders in naturally aging mice. We found that TWK10 administration attenuated aging-related muscle weakness by improving muscle quality and increasing muscle glycogen levels. TWK10 slowed down the progression of age-related bone loss and increased trabecular numbers in the murine femur. TWK10 treatment also significantly attenuated the cognitive impairment of aged mice by shortening their MWM test mean escape latencies. Moreover, gut microbiota analysis by next-generation sequencing (NGS) of the 16S rRNA gene demonstrated that the gut microbial composition was significantly altered following TWK10 administration, reducing the natural accumulation of pathogenic organisms (such as *Enterobacteriaceae* and *Enterococcaceae*) that occurs with age, while boosting the abundances of beneficial SCFA-producing bacteria.

Conclusively, we have confirmed that *Lactobacillus plantarum* TWK10 could be considered as a potential therapeutic agent to promote healthy aging by attenuating aging-related disorders and modulating the imbalance of gut microbiota. Future studies are needed to validate the results obtained using animal models in a clinical setting, prior to confirming the health-promoting benefits of TWK10 in humans.

## Data Availability Statement

The datasets presented in this study can be found in online repositories. The names of the repository/repositories and accession number(s) can be found at: https://www.ncbi.nlm.nih.gov/bioproject/PRJNA726848.

## Ethics Statement

The animal study was reviewed and approved by Institutional Animal Care and Use Committee (IACUC) of National Taiwan Sport University, Taoyuan City, Taiwan.

## Author Contributions

C-CL and Y-CL analyzed and interpreted the data and wrote the manuscript. M-CL, H-YH, and S-YC conducted the experiments and collected data. S-LY, J-SL, and C-CH designed and oversaw the study. KW interpreted the data and edited the manuscript. All authors read and approved the final manuscript.

## Conflict of Interest

C-CL, Y-CL, H-YH, S-YC, S-LY, J-SL, and KW are employed by SYNBIO TECH INC. The remaining authors declare that the research was conducted in the absence of any commercial or financial relationships that could be construed as a potential conflict of interest.

## Publisher's Note

All claims expressed in this article are solely those of the authors and do not necessarily represent those of their affiliated organizations, or those of the publisher, the editors and the reviewers. Any product that may be evaluated in this article, or claim that may be made by its manufacturer, is not guaranteed or endorsed by the publisher.
