# Supplementary material for: Lactobacillus plantarum TWK10 Attenuates Aging-Associated Muscle Weakness, Bone Loss, and Cognitive Impairment by Modulating the Gut Microbiome in Mice
Source: Front Nutr. 2021 Oct 13;8:708096. doi: 10.3389/fnut.2021.708096 (PMC8548577; doi:10.3389/fnut.2021.708096)
Supplement: Supplementary file 2 [file Data_Sheet_1.PDF]

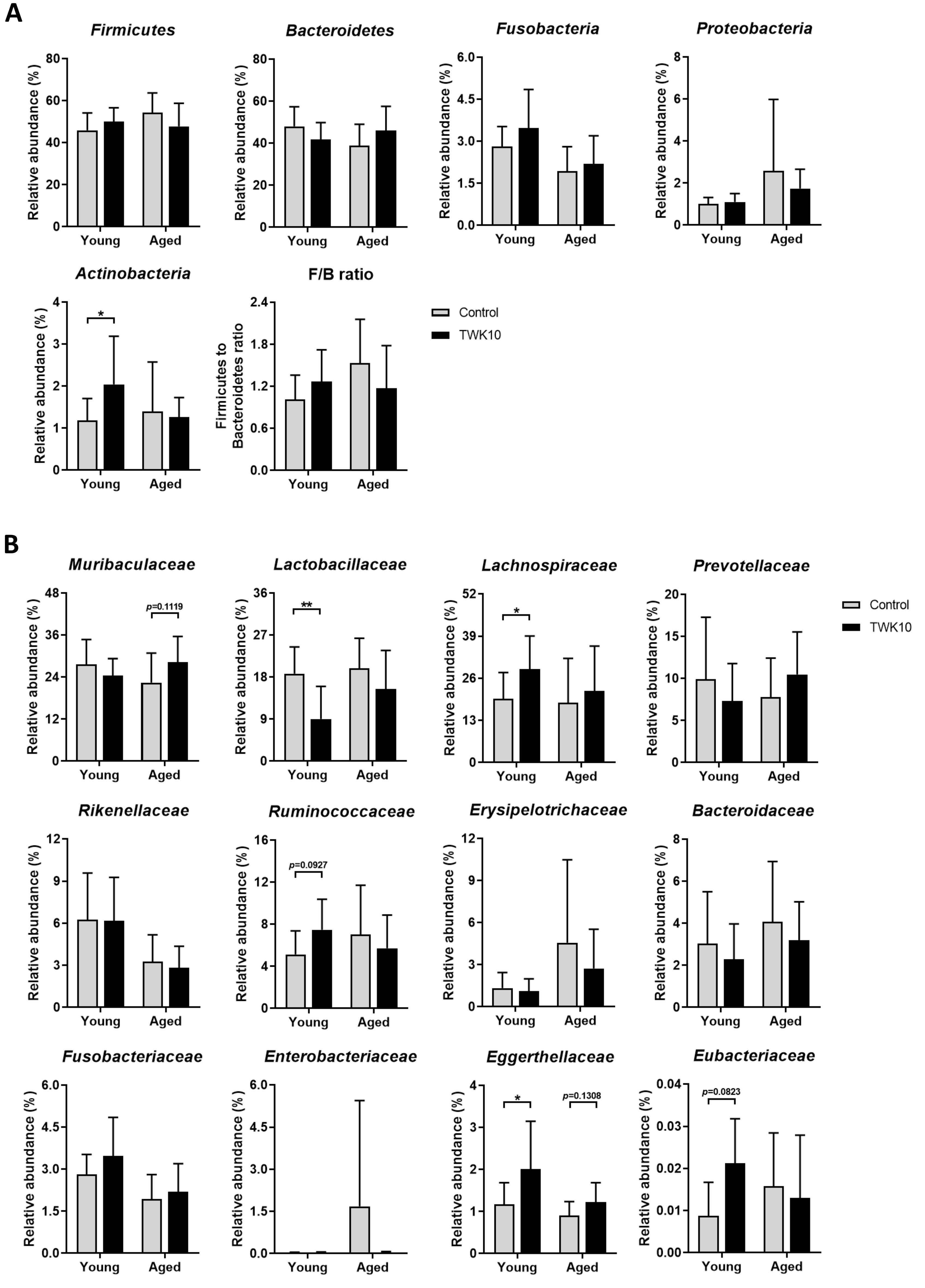

Figure S1. Quantifications of major gut microbiota at the phylum and family levels. (A) Quantification of major gut bacteria at phylum level. (B) Quantification of gut bacteria at family level. Data are represented as mean ± SD. Treatment effect was statistically analyzed by Mann-Whitney *U* test, *$P < 0.05$, **$P < 0.01$.

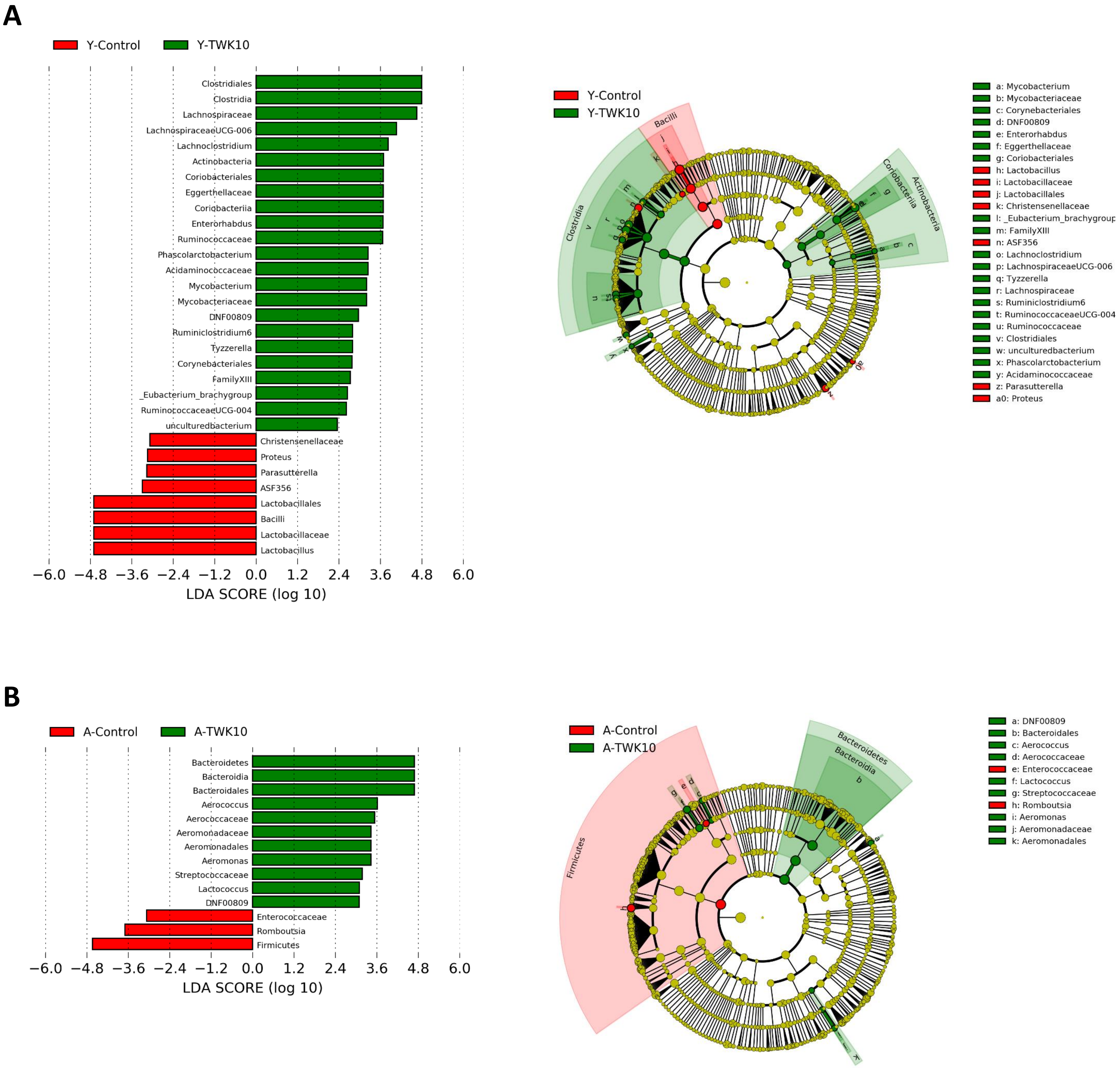

Figure S2. LEfSe analysis identifying the most differentially abundant taxa between control and TWK10-administered mice groups. Linear discriminant analysis (LDA) coupled with effect size (LEfSe) identified differentially abundant bacterial taxa at phylum, class, family and genus levels between vehicle control and mice with TWK10 administration. Cladograms are derived from LEfSe analysis of differential gut microbial taxa. The central point denotes the root of the tree of bacteria and expanded to each ring representing the next lower taxonomic level from phylum to genus. Each circle's diameter represents the relative abundance of the taxon in gut microbial community. (A) Differences in the gut microbial communities of young mice groups. (B) Differences in the gut microbial communities of aged mice groups. Control-enriched taxa are indicated with positive LDA score (green), and taxa enriched in TWK10-administered are shown in negative score (red). Only LDA score ≥ 2.0 were shown and considered significant.

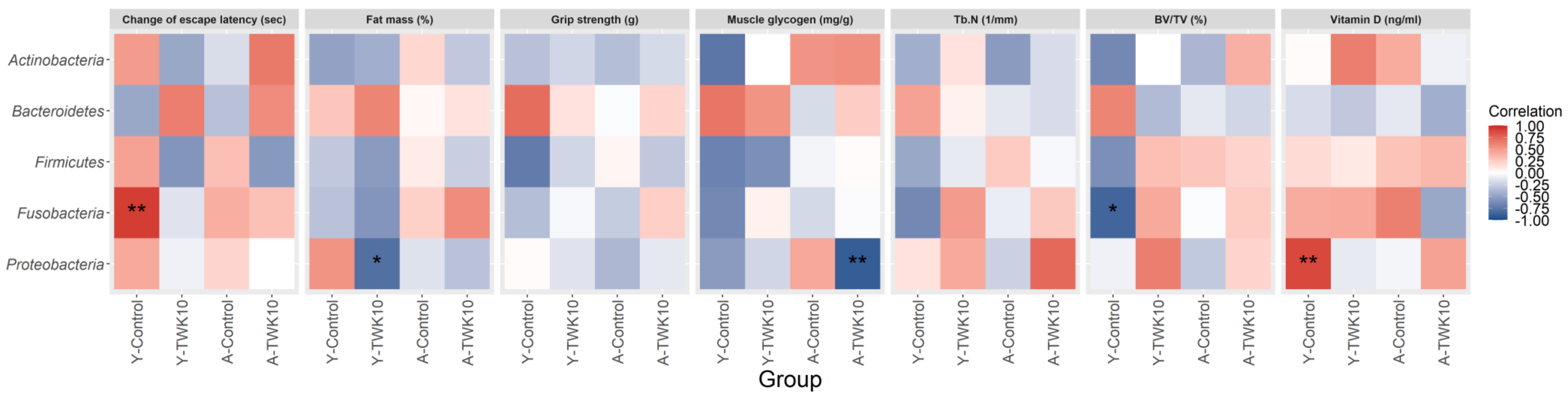

Figure S3. Relationship between gut microbiota and altered age-related host phenotypic features. Spearman's correlation analysis was performed to investigate the correlations between the relative abundances of top five phyla and the values of seven altered aged-related host phenotypic features selected from the differential analysis between Y-Control and Y-TWK10 or A-Control and A-TWK10 groups. Red squares indicate positive correlations and blue squares indicate negative correlations. *$P < 0.05$,**$P < 0.01$.

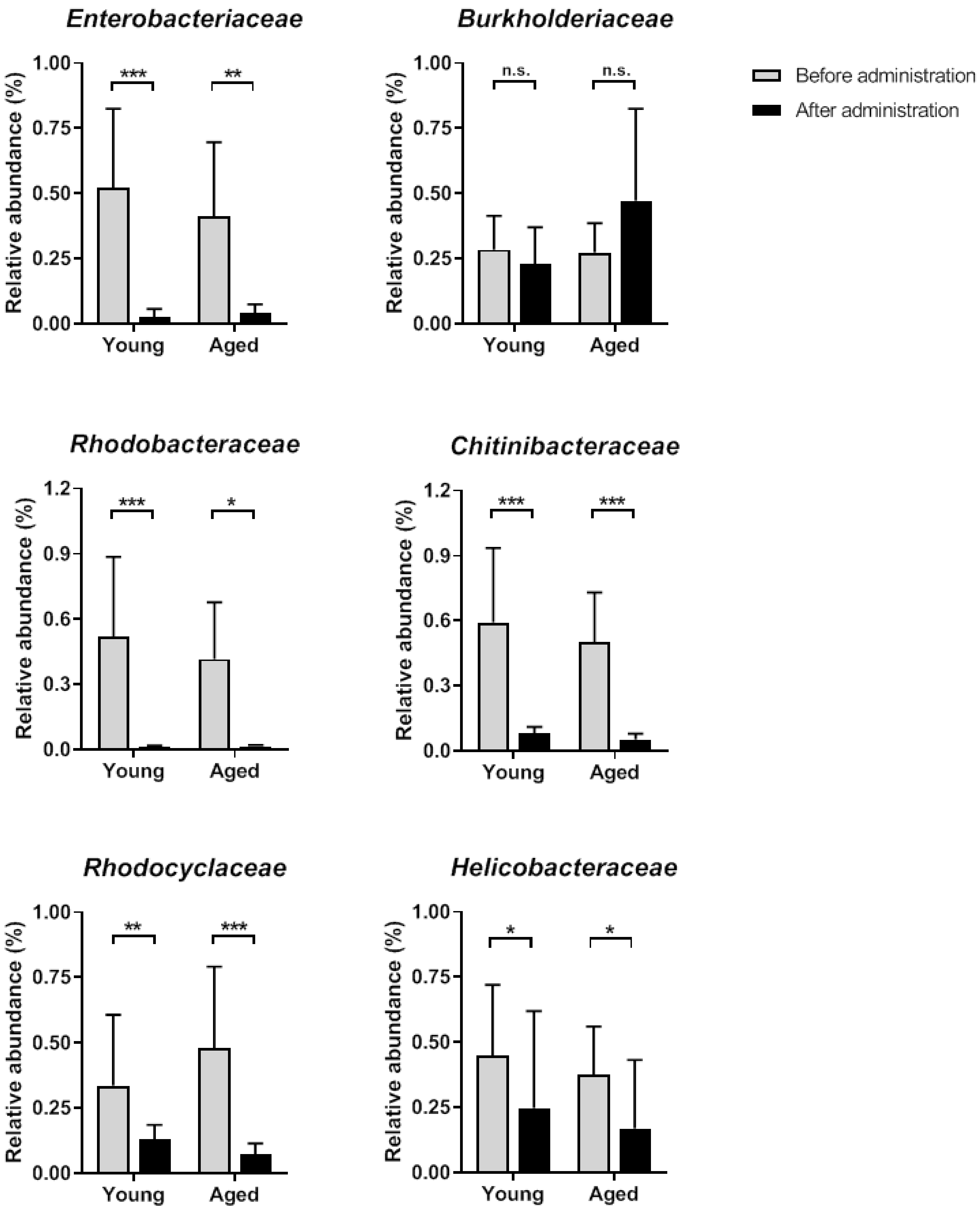

Figure S4. Changes in the relative abundances (%) of major bacterial families belonging to the phylum *Proteobacteria* between before and after administration of TWK10 for eight weeks in young and aged mice. Effect of TWK10 administration was statistically analyzed by Mann-Whitney *U*-test.

**P* < 0.05, ***P* < 0.01, ****P* < 0.001.

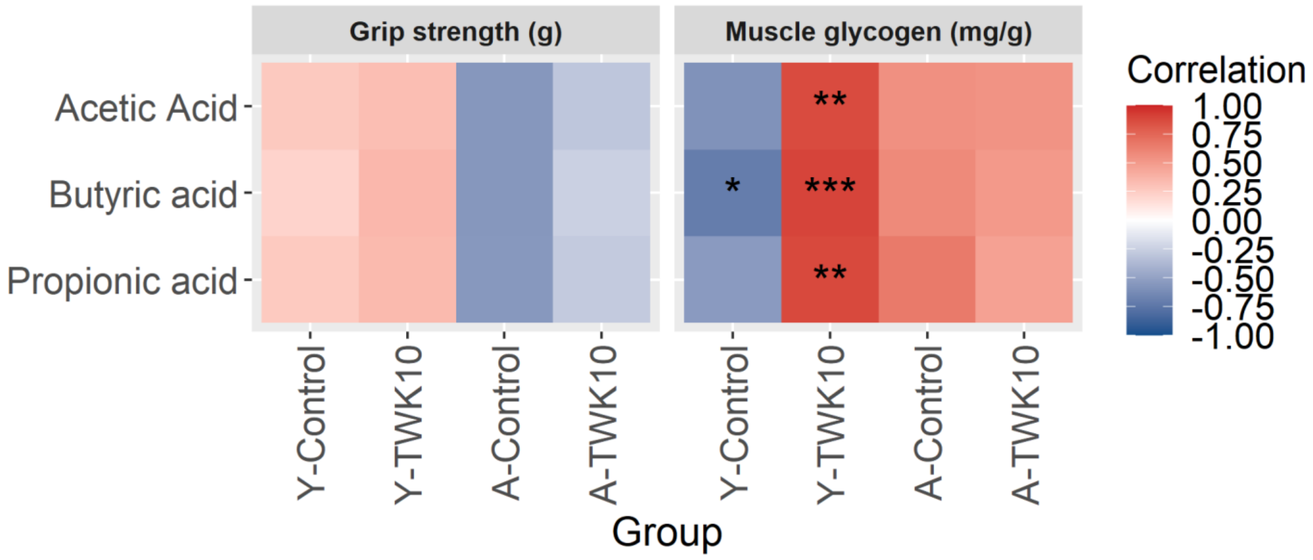

Figure S5. TWK10-mediated improvements of SCFA production and correlate with beneficial effects of muscle quality. Heat map representation of the Pearson's r correlation coefficient between cecal SCFAs and performance of grip strength, and cecal SCFAs and muscle glycogen levels. *$P < 0.05$, ***$P < 0.001$.
